# Facile synthesis of ultrahigh-surface-area hollow carbon nanospheres for enhanced adsorption and energy storage

**DOI:** 10.1038/ncomms8221

**Published:** 2015-06-15

**Authors:** Fei Xu, Zhiwei Tang, Siqi Huang, Luyi Chen, Yeru Liang, Weicong Mai, Hui Zhong, Ruowen Fu, Dingcai Wu

**Affiliations:** 1 Materials Science Institute, PCFM Lab and GDHPPC Lab, School of Chemistry and Chemical Engineering, Sun Yat-sen University, Guangzhou 510275, China

## Abstract

Exceptionally large surface area and well-defined nanostructure are both critical in the field of nanoporous carbons for challenging energy and environmental issues. The pursuit of ultrahigh surface area while maintaining definite nanostructure remains a formidable challenge because extensive creation of pores will undoubtedly give rise to the damage of nanostructures, especially below 100 nm. Here we report that high surface area of up to 3,022 m^2^ g^−1^ can be achieved for hollow carbon nanospheres with an outer diameter of 69 nm by a simple carbonization procedure with carefully selected carbon precursors and carbonization conditions. The tailor-made pore structure of hollow carbon nanospheres enables target-oriented applications, as exemplified by their enhanced adsorption capability towards organic vapours, and electrochemical performances as electrodes for supercapacitors and sulphur host materials for lithium–sulphur batteries. The facile approach may open the doors for preparation of highly porous carbons with desired nanostructure for numerous applications.

## Abstract (web summary)

Well-defined, large surface area nanostructures are promising functional materials but can be difficult to fabricate. Here the authors show how to prepare ultrahigh-surface-area hollow carbon nanospheres, via a controlled carbonization route, and assess their organic vapour adsorption and electrochemical performance.

## Graphical abstract

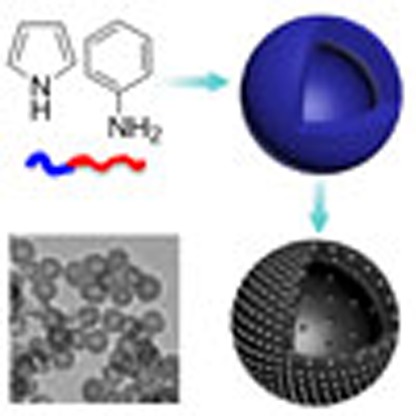

Carbon materials, including carbon nanotubes^1^, graphene^2^ and nanoporous carbons^3^, have been investigated extensively to find utility in many practical applications. Among these, nanoporous carbons with unique features such as large specific surface area (SSA), high porosity and controlled nanostructures are attracting significant scientific and technological interest^3^,^4^,^5^,^6^,^7^; these intriguing structural characteristics permit their use in various applications including adsorption media, chromatographic separation systems, catalyst supports, nanoreactors and electrodes for batteries and supercapacitors. Exploring exceptionally high SSAs with well-defined nanostructures has been a long-pursued goal for the development of the state-of-the-art nanoporous carbons, which may provide new opportunities in these emerging applications and further expand their application scopes. Many strategies have been reported to prepare porous carbons with high SSAs, including activation^8^,^9^,^10^,^11^, direct carbonization of crosslinked/conjugated polymers^12^,^13^ and metal–organic frameworks (MOFs)^14^,^15^,^16^, high-temperature chlorination of carbide materials^17^,^18^,^19^, self-assembly of supramolecular complexes (for example, phenolic resins with block copolymer templates)^20^,^21^ and nanocasting strategy with hard templates (for example, nanoporous silica and zeolites)^3^,^4^,^22^. Among various strategies, chemical activation (for example, KOH) is recognized as a well-established method for generating highly porous structures with very large SSAs (for example, 3,000 m^2^ g^−1^). However, extensive creation of pores will undoubtedly cause the collapse of their nanostructures^23^. Thus, the development of ultrahigh SSA while maintaining definite nanostructures remains a formidable challenge.

Hollow carbon nanospheres (HCNs), a class of intriguing nanoporous carbon materials, have received much research interest by virtue of the special shape, low density and large interior void space fraction, allowing their many potential applications^24^,^25^,^26^,^27^,^28^,^29^. The key to the success in applications strongly depends on the ability to design well-defined HCNs, coupled with highly porous structures. In general, templating strategy involving hard/soft templates could be the most frequently used technique to prepare HCNs, which involves coating carbon precursor onto a predesigned solid spherical core template, carbonization, activation sometimes and removal of the template (Fig. 1a)^24^,^25^,^26^. This strategy allows for fine control of hollow cores by selection of different template sizes. Nevertheless, surface modification of template is generally required for uniform coating. The removal of template is often needed for hard templating, which seems time-consuming, severe and harmful to the environment (for example, silica template with hydrofluoric acid etching), whereas for soft templating, the thermo-decomposable soft template can be directly removed during carbonization. Furthermore, it is very difficult to control the diameter of HCNs below 100 nm, a size that is essential to many valuable nanoscale effects. This is because small core templates tend to aggregate seriously, leading to ill-defined hollow structures. More importantly, the SSAs of HCNs reported so far are relatively low (typically ≤1,800 m^2^ g^−1^; Supplementary Table 1). This might be attributed to the collapse of the hollow structure upon intensive pore-making treatment. The low SSAs not only render HCNs at a disadvantage in gas sorption but also deteriorate their performances in energy storage, guest encapsulation, catalysis and so on. Thus, the nanospherical diameter below 100 nm and SSA beyond 1,800 m^2^ g^−1^ represent a largely unfilled gap for HCNs.

Herein, we propose a facile method to develop a class of well-defined HCNs that possess the highest surface area and lowest uniform nano-diameter reported to date, to the best of our knowledge. As shown in Fig. 1b, HCNs are fabricated by simple carbonization of polyaniline-co-polypyrrole (PACP) hollow spheres without tedious templating and activation procedures. This is realized by careful selection of carbon precursors and carbonization conditions. The robust conjugated structure of the PACP carbon precursor ensures sufficient carbonization of the framework with inherited nanostructure, while simultaneous choice of aniline and pyrrole comonomers enables the formation of a hollow structure in the presence of Triton X-100. The SSA can be tailored over a broad range by varying thermal treatment conditions, such as carbonization temperature and time, and heating rate, whereas the nanospherical morphology is well retained. The highest SSA reaches 3,022 m^2^ g^−1^ with a uniform diameter as low as 69 nm. The unusual integration of ultrahigh porous structure and well-defined hollow spherical morphology in the nanometre range is found to provide an unprecedented opportunity to boost the performances of HCNs to a new stage. For example, the HCNs demonstrate high uptake capacities towards environment-concerned organic vapours and serve well as electrode materials for supercapacitive energy storage and cathode host materials for lithium–sulphur (Li–S) batteries. These findings may open a new avenue for the design and construction of highly porous yet well-defined nanostructured carbon materials for challenging energy and environmental issues.

## Results

### Fabrication and nanostructure control of HCNs

A facile template-free strategy, which includes large-scale fabrication of HCN precursors and subsequent thermal treatment for carbonization, was developed to prepare high-level HCNs. Briefly, the carbon precursors, hollow PACP nanospheres (Fig. 2a), were prepared using the strategy of confined interfacial copolymerization of aniline and pyrrole in the presence of Triton X-100 micelle^30^. Then, the hollow PACP nanospheres were directly carbonized in a furnace under protection of an inert gas to readily obtain the target HCNs. The key to realize highly porous structures is the utilization of the robust conjugated polymeric precursor that permits sufficient framework carbonizability and nanostructure inheritability, regardless of the rigorously applied carbonization conditions. The obtained HCNs exhibit a well-defined nanospherical morphology (Fig. 2b and Supplementary Fig. 1) with a narrow particle size distribution even after harsh thermal treatment (Fig. 2c). HCNs present smaller diameters than their precursor PACP (Fig. 2a), resulting from the framework shrinkage during carbonization. For example, HCN-900-20H2R obtained at 900 °C for 20 h with a heating rate of 2 °C min^−1^ has an outer diameter of 69 nm (Fig. 2c), which is obviously lower than that of PACP (106 nm; Fig. 2a and Supplementary Fig. 2). Such a diameter, to our knowledge, is the smallest one reported so far for HCNs with uniform morphology (normally 100–900 nm; Supplementary Table 1). HCN-900-20H2R shows a clear single hollow core of 26 nm in diameter (Fig. 2e,f), indicating that the shell thickness is 21.5 nm. Other HCNs (for example, HCN-900-10H5R), obtained at less rigorous carbonization conditions, certainly possess the uniform hollow nanosphereric morphology (Supplementary Fig. 2b–d). HCNs have good electrical conductivity. For example, HCN-900-10H5R exhibits a high conductivity of 3.5 S cm^−1^, which is larger than those of commercial activated carbons such as YP-50 (0.6 S cm^−1^) and SPC-01 (1.07 S cm^−1^), and many other nanocarbons 31,32,33,34. HCN-900-10H5R presents a tapping density of 0.25 g cm^−3^, a value comparable to those for commercial activated carbons such as SPC-01 (0.24 g cm^−3^) and YP-50 (0.35 g cm^−3^). In addition, HCNs are found to have nitrogen-containing functional groups. The nitrogen content for HCN-900-10H5R and HCN-800-3H2R was measured to be 2.55 and 7.92 wt%. The nitrogen-containing functional groups can be mainly assigned to pyridinic N (N-6) and quaternary N (N-Q) (Supplementary Fig. 3).

Nitrogen adsorption experiment was performed to examine the pore characteristics of HCNs (Fig. 3). The nitrogen adsorption–desorption isotherm of HCN-900-20H2R exhibits characteristics of type I/IV according to the classification of the International Union of Pure and Applied Chemistry. A very high nitrogen uptake at low relative pressure demonstrates the existence of tremendous nanopores within the shell, whereas the hysteresis loop at high relative pressure indicates the presence of mesopores. For HCN-900-20H2R, the Brunauer–Emmett–Teller (BET) surface area (S_BET_) is calculated to be as high as 3,022 m^2^ g^−1^, which mainly originates from numerous nanopores with a maximum peak at 2.5 nm in its carbon shell (the inset in Fig. 3a). To the best of our knowledge, this is the highest SSA for HCNs reported so far (generally 200–1,800 m^2^ g^−1^; Supplementary Table 1). In sharp contrast, the carbon precursor PACP, only shows S_BET_ of 33 m^2^ g^−1^ (Fig. 3a). This result demonstrates that the nanopores in the shell of HCNs are generated during carbonization treatment. This is because the resulting carbon shell are composed of turbostratic carbon sheets and clusters with a microcrystalline plane crystal size of 0.78 nm (Supplementary Figs 4 and 5, and Supplementary Table 2), and their disordered packing leads to free volume and porosity^35^,^36^.

The pore structure of this class of HCNs can be easily controlled by tuning the carbonization conditions, such as temperature, time and heating rate (Supplementary Fig. 6 and Supplementary Table 3). Both increasing the carbonization temperature and time lead to the increase of porosity, whereas the role of heating rate is not so significant (Fig. 3b–d). For example, S_BET_ increases from 219 to 936 m^2^ g^−1^ when the carbonization temperature goes up from 400 to 1,000 °C at a heating rate of 2 °C min^−1^. This feature is different from some other carbon precursors such as MOFs, where increasing the carbonization temperature will give rise to lower surface areas owing to the collapse of nanoporous structure caused by graphitization^14^,^37^,^38^. It is also different from the relatively low-crosslinked polymeric resins, which undergo sinter, fusion and collapse of polymeric networks and nanostructure during the initial semi-carbonization^39^. With extending the carbonization time from 3 to 20 h, the S_BET_ increases drastically from 858 to 3,022 m^2^ g^−1^, while increasing the heating rate from 2 to 10 °C min^−1^ gives a slight increase from 1,595 to 2,050 m^2^ g^−1^. In spite of the distinct porous structures, the uniform nanospherical morphology retains intact (Supplementary Fig. 1), demonstrating the excellent inheritability of the nanostructure by using the robust conjugated carbon precursor.

### Organic vapour adsorption performances

The exceptionally large surface areas together with the adjustable pore structures make HCNs very attractive candidates for adsorption applications. The adsorption properties towards organic vapours, such as methanol and toluene, were investigated, which are of concern to the environment. As indicated in Fig. 4, the highest adsorption amounts of toluene and methanol at room temperature for HCN-900-20H2R are up to 1,500 and 1,230 mg g^−1^, respectively, at a relative vapour pressure of 0.9. The excellent adsorption performances are very competitive as compared with those of so many other porous materials, such as porous carbons (243–641 and 456–710 mg g^−1^ for methanol and toluene, respectively), nanoporous polymers (289–934 and 780–1,357 mg g^−1^ for methanol and toluene, respectively) and MOFs (100–480 and 125–1,285 mg g^−1^ for methanol and toluene, respectively; Supplementary Fig. 7 and Supplementary Table 4). Such an adsorption advantage enables great potential for their further environmental applications. Moreover, the adsorption capability can be tailored by using HCNs with different surface areas (Fig. 4); that is to say, one can choose an appropriate HCN as one wishes for different adsorption purposes to realize a target-oriented use. For example, with increasing the surface area of HCNs from 858, 2,095 to 3,022 m^2^ g^−1^, the adsorption amount can be tuned from 368, 925 to 1,500 mg g^−1^ for toluene vapour and 271, 779 to 1,230 mg g^−1^ for methanol vapour, respectively. Generally, it is believed that extensive exploration of the nanopores undoubtedly increases the number of pores, but at the expense of forming a great deal of inaccessible pore surface, namely, the decreased surface utilization efficiency (i.e., the adsorption amount divided by the SSA here). To our delight, the surface utilization efficiency of these HCNs keeps almost constant or even increases slightly for those with higher SSAs (Supplementary Fig. 8). These results unambiguously indicate that the effective pore-making strategy allows the highly accessible pore surface areas. In addition, HCNs demonstrate an excellent regeneration property, judging from the adsorption retention ratio of almost 100% over 10 cycles (Supplementary Fig. 9). All in all, the resulting HCNs are tolerant to different desired adsorption utilizations and show wonderful adsorption properties towards organic vapours, which would have potential use to eliminate harmful organic vapours in the environment.

### Supercapacitive performances

Electric double-layer capacitors (EDLCs) or supercapacitors, featured by the high power density, rapid charging period and long-cycling life, represent an emerging class of power sources for portable electronic devices^6^,^7^,^40^. It remains a long-standing goal to develop carbon electrodes having both high capacitance, strongly related to the availability of ion-accessible micropores, and high ion transport rate, closely associated with the availability of meso-/macropores and the size of carbon particles^11^,^41^,^42^. In this respect, HCNs will be very promising electrode materials for EDLCs because they bear well-developed porous structures, well-defined hollow nanosphereric morphology and nitrogen-containing functional groups. As a proof-of-concept demonstration, the HCN-900-10H5R was evaluated with a two-electrode symmetrical cell in 6 M KOH aqueous electrolyte. The cyclic voltammetry (CV) curves exhibit rectangular shapes and the galvanostatic charge–discharge curves display typical triangular profiles (Fig. 5a,b). These results clearly highlight the superior supercapacitive performances, benefitting from their unique porous nanostructures. The tremendous small-sized nanopores on the carbon shells can strongly adsorb a large quantity of electrolyte ions for high capacitances, whereas the uniform nanospheres (<100 nm) with hollow cores can significantly decrease the ion diffusion length (half of the shell thickness, for example, 12 nm for HCN-900-10H5R; Supplementary Fig. 2; ref. 43). Moreover, the nitrogen-containing functional groups are also beneficial to the superior supercapacitive performances by improving electrolyte wettability and electrical conductivity and providing additional pseudocapacitance^6^,^7^. In sharp contrast, the nanopores in the activated carbon AC are mainly located on the surface of micron-/millimetre-scaled carbon particles (for example, >10 μm; ref. 41). This causes greatly retarded mass transfer, and thereby places a great barrier for applications in high power EDLCs. Therefore, the CV curves of AC distort much seriously, especially with increasing the sweeping rate (Supplementary Fig. 10), and the galvanostatic discharge curves present a notable voltage drop at high current densities (Supplementary Fig. 11).

The specific capacitance of HCN-900-10H5R is 201 F g^−1^ at 5 mV s^−1^ and retains 169 F g^−1^ even at a high sweep rate of 500 mV s^−1^ with a capacitance retention ration of 84% (Fig. 5c,d). Also, the capacitance of 203 F g^−1^ at 0.1 A g^−1^ is obtained, with a slight decrease to 180 F g^−1^ while 10 time increase of the current density from galvanostatic charge–discharge tests (Supplementary Fig. 12). The corresponding capacitance retention ratios far exceed those of the commercial AC (Fig. 5d) and many other reported high-rate porous carbons, including HCNs, ordered mesoporous carbons and hierarchical porous carbons (Supplementary Table 5). Ragone plot shows that an energy density of 9.17 Wh kg^−1^ is achieved, much higher than the common carbon electrode of 5 Wh kg^−1^ (Supplementary Fig. 13). The facilitated ion transport of HCN-900-10H5R can also be confirmed from the electrochemical impedance spectra, in which the charge transfer resistance is much lower than that of AC (Fig. 5e). HCN-900-10H5R exhibits very high cycle stability with little degradation at a current density of 1 A g^−1^ over 5,000 cycles (Fig. 5f), indicating a robust architecture of the hollow nanospherical carbon framework during the charging–discharging process. Our HCNs demonstrate the unique combination of high specific capacitance, excellent stability and outstanding capacitance retention ratio, which paves the way for realizing high-energy-density EDLCs without sacrificing the power density.

### Li–S battery performances

Our HCNs can be also applied as high-performance cathode host materials to immobilize sulphur for Li–S batteries. Element sulphur as cathode possesses high theoretical capacity (1,675 mA hg^−1^) and energy density (2,567 Wh kg^−1^), much higher than those of conventional inorganic metal-based lithium-ion batteries^44^,^45^,^46^,^47^. However, several major issues have to be overcome before Li–S batteries can find their widespread practical application. They include poor conductivity of sulphur and discharge product Li_2_S, serious dissolution of intermediate polysulphides with a shuttling phenomenon, and large volumetric expansion (76%) upon lithiation^46^,^47^. These problems result in poor cyclability, low coulombic efficiency and deteriorated rate capability. Motivated by the pioneer work by Nazar and colleagues^48^ using mesoporous carbon particles to encapsulate sulphur, exciting progress has recently been made in trapping polysulphide species by porous carbons. HCNs, in this respect, are particularly attracting as host materials for sulphur accommodation^27^,^49^,^50^,^51^. Sulphur could be encapsulated and sequestered in their conductive nanoporous shell, which is beneficial for enhancing charge transfer and accommodating volume expansion during redox cycling. Moreover, the sulphur content in nanocomposites can be controlled by choosing HCNs with different pore volumes, so as to tailor the overall capacity (TGA curves; Supplementary Fig. 14). Melt-diffusion method is used to impregnate sulphur into the nanopores of HCNs. After sulphur impregnation, the S_BET_ and pore volume of the resulting HCN-900-10H5R/S nanocomposite drastically decrease (Supplementary Fig. 15), and no any discernible sulphur particles is detected outside the HCN-900-10H5R (Fig. 6a,b,e). TEM images of the HCN-900-10H5R/S composite indicate that the hollow cores of HCN-900-10H5R are not occupied by sulphur (Fig. 6b,e), revealing that the majority of sulphur is impregnated into the nanopores of shells of HCN-900-10H5R. Elemental mapping images of C and S demonstrate the uniform distribution of sulphur (Fig. 6c,d). The diffraction signals assignable to crystalline sulphur disappear in the X-ray diffraction (XRD) profiles (Fig. 6f), and no signal below 500 cm^−1^ is found in HCN-900-10H5R/S in Raman spectra (Fig. 6g), further confirming that sulphur is in a homogeneously dispersed state^48^. The uniformly encapsulated sulphur exists in the form of elemental sulphur, judging from the almost unchanged peaks at 163.8 and 164.9 eV in X-ray photoelectron spectroscopy (XPS; Supplementary Fig. 16).

Fig. 7a presents galvanostatic discharge–charge curves at a rate of 0.5 C for 100 cycles (*n* C represents full delivery of the theoretical capacity in 1/*n* h, 1 C=1,675 mA g^−1^). Two plateaus at 2.3 and 2.0 V are observed in the discharge curves, which are assigned to the reduction of cyclic S_8_ to long-chain lithium polysulphides (Li_2_S_*x*_, 4≤*x*≤8) and further reduction to short-chain lithium polysulphides (Li_2_S_*x*_, *x*≤3) and Li_2_S, respectively^52^,^53^,^54^. During the charging process, only one plateau at 2.4 V assignable to the oxidation of Li_2_S is observed. The nanostructured composite shows a capacity of 1,043 mAh g^−1^ at the initial discharge, whereas simply mixing the sulphur with HCN-900-10H5R gives only a low capacity of 652 mAh g^−1^ (Fig. 7b and Supplementary Fig. 17). The plateaus retain their shapes after 100 cycles, and the coulombic efficiency keeps at 100% throughout 100 cycles, implying that only little fraction of polysulphide anions diffuse into the electrolyte due to efficient sequestering. The capacity is rather stable upon 100 cycles with a slight decrease of 0.07% per cycle and capacity retention ratio of 93%. This value is superior to that of previously reported HCNs and many other porous carbon substrates (Supplementary Table 6). HCN-900-10H5R/S electrode still keeps the spherical morphology with little change in surface structure after 100 cycles (Supplementary Fig. 18). The stable performance can be also reflected from the electrochemical impedance spectra, where the charge transfer resistance, determined by the semicircle at high-frequency region^41^, is quite stable upon 100 cycles, except for a larger resistance in the initial cycle because of the electrolyte penetration effect for activation (Supplementary Fig. 19 and refs 55, 56).

The rate capability was also evaluated from 0.2 to 2 C with 10 cycles at each. After an initial discharge capacity of 1,240 mAh g^−1^ at 0.2 C, the capacity is found to stabilize at 1,167 mAh g^−1^. Further cycling at 0.5, 1 and 2 C delivers high reversible capacities of 1,026, 965, 655 mAh g^−1^, respectively (Fig. 7c,d). When the C rate is switched abruptly from 2 to 1 C again, the original capacity is largely recovered (Fig. 7c), revealing the robustness and stability of the HCN-900-10H5R/S cathode structure. These results demonstrate the excellent high rate capabilities, which are comparable to or even better than some of previously reported carbon/sulphur composite cathodes such as double-shelled hollow carbon spheres (1,100–350 mAh g^−1^ at 0.1–1 C; ref. 51), mesoporous carbon nanospheres (850–400 mAh g^−1^ at 0.09–1.8 C; ref. 21), ordered meso@microporous carbons (1,182–605 mAh g^−1^ at 0.1–2 C; ref. 57) and so on. In addition, the HCN-900-10H5R/S nanoarchitecture still exhibits stable cycling performance over 500 cycles at 1 C after stopping the rate performance test for one month (Fig. 7e). The discharge capacity starts at 647 mAh g^−1^ and shows a gradual increase to 842 mAh g^−1^ after the first 12 cycles owing to the activation effect for electrolyte diffusion, which is in agreement with the observation of electrochemical impedance spectroscopy curves (Supplementary Fig. 19). The cell retains a high capacity of 629 mAh g^−1^ even after 500 cycles, and the coulombic efficiency keeps at 100–103%. It is noted that such a long-term stability is a very attracting result for a Li–S secondary battery using porous carbon/sulphur composites without further modification (Supplementary Table 6). For a given carbon, increasing the sulphur content will increase the overall capacity based on carbon/sulphur composite, but at the expense of sacrificing the sulphur utilization and cycle stability. This is ascribed to the presence of unconfined sulphur and the decrease of composite conductivity. Surprisingly, the HCNs, bearing different pore volumes, permit the encapsulation of sulphur content from 43 to 67% to maximize the overall capacities (Supplementary Fig. 14), without sacrificing the cycle stability (Supplementary Figs 20 and 21). The highest overall capacity can reach reversible 756 mAh g^−1^ at 0.5 C with a stable cycling performance (Supplementary Fig. 21).

## Discussion

To better reveal the important factors for the formation of ultrahigh-surface-area HCNs, several experiments were carried out for contrast. On one hand, the robust conjugated polymeric framework is very decisive to retain the hollow nanospherical morphology under harsh carbonization treatment. As a demonstration, crosslinked polystyrene-based hollow nanosphere is used as the HCN precursor^58^. It was prepared by a procedure of direct templating and subsequent hypercrosslinking, in which silica serves as core template and crosslinked polystyrene as shell. A typical carbonization treatment of 3 h gives an intact hollow spherical structure; however, extending the carbonization time to the same 20 h as that of HCN-900-20H2R fails to keep the hollow structure (Supplementary Fig. 22). On the other hand, the use of the sole aniline or pyrrole cannot obtain uniform carbon hollow structure owing to failure of construction of polymeric hollow structure (Supplementary Fig. 23). These results highlight the importance of the choice of carbon precursors, which may offer a benchmark for preparing well-defined yet highly porous nanostructured carbon materials via using other similar carbon precursors.

**Figure 1:**
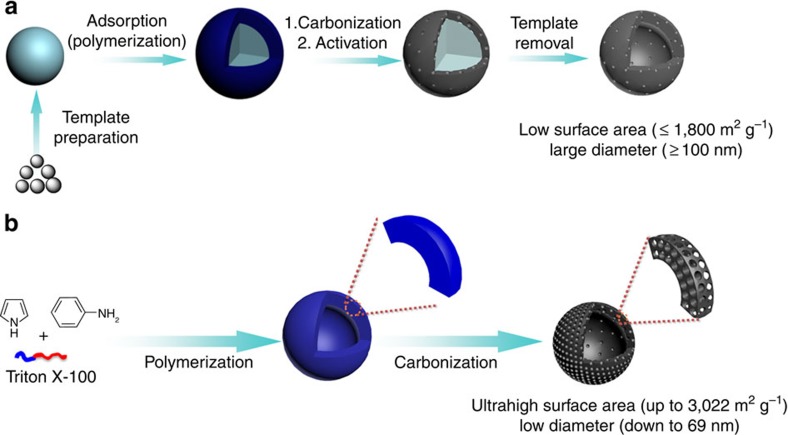
Schematic illustration of preparation of HCNs. (**a**) Fabrication of conventional HCNs by a tedious templating method. This method usually includes preparation of a predesigned core template, adsorption and polymerization of raw materials of polymeric shell, carbonization, additional activation sometimes and removal of hard templates. For the as-obtained conventional HCNs, their BET surface areas are very difficult to exceed 1,800 m^2^ g^−1^ and their diameters are very hard to decrease down to 100 nm. (**b**) Design and fabrication of novel HCNs through a facile procedure without any tedious templating and activation steps. This is achieved via the simple carbonization treatment of well-orchestrated PACP hollow spheres. For the resulting new HCNs, their BET surface areas can be up to 3,022 m^2^ g^−1^ with a uniform diameter as low as 69 nm.

**Figure 2:**
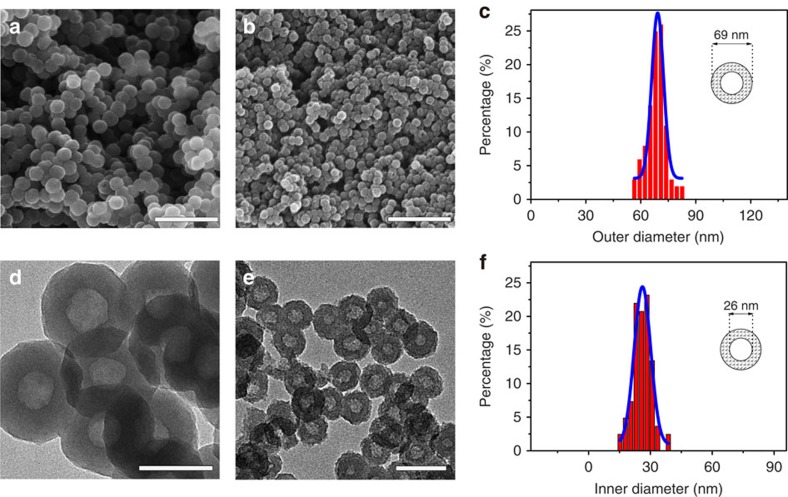
Nanomorphologies of HCNs. Scanning electron microscope (SEM) images of (**a**) PACP and (**b**) HCN-900-20H2R; transmission electron microscope (TEM) images of (**d**) PACP and (**e**) HCN-900-20H2R; (**c**) outer and (**f**) inner diameter distribution histograms of HCN-900-20H2R from analysis of SEM and TEM images, respectively. Scale bars, 500 nm (**a**,**b**), 100 nm (**d**) and 200 nm (**e**).

**Figure 3:**
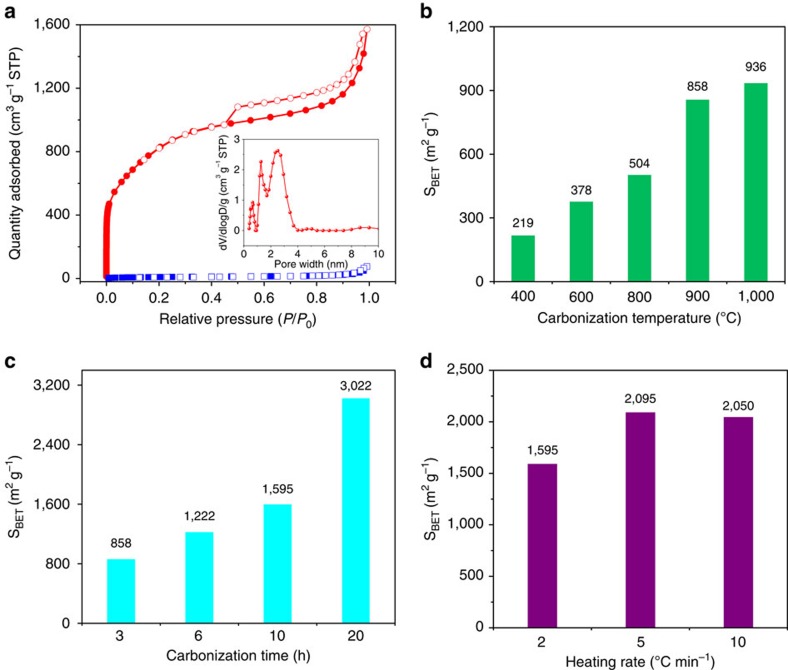
Pore structures of HCNs. (**a**) N_2_ adsorption–desorption isotherms of HCN-900-20H2R (red) and its PACP carbon precursor (blue); the inset shows the density functional theory pore size distribution of nanopores in the shell for HCN-900-20H2R. S_BET_ of HCNs obtained at various carbonization conditions, including (**b**) carbonization temperatures, (**c**) carbonization times and (**d**) heating rates.

**Figure 4:**
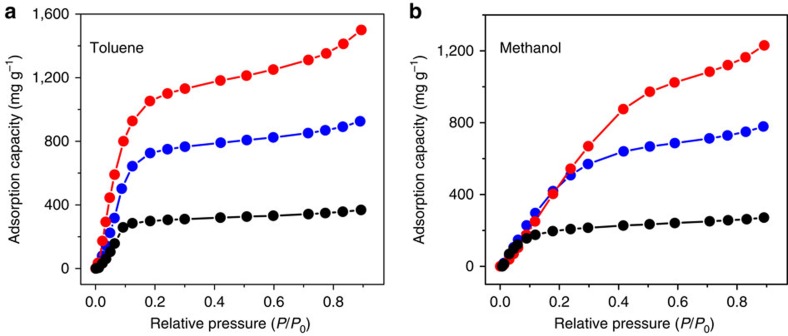
Organic vapour adsorption performances of HCNs. Adsorption capacity towards (**a**) toluene and (**b**) methanol vapours at various relative pressures for HCN-900-20H2R (red), HCN-900-10H5R (blue) and HCN-900-3H2R (black).

**Figure 5:**
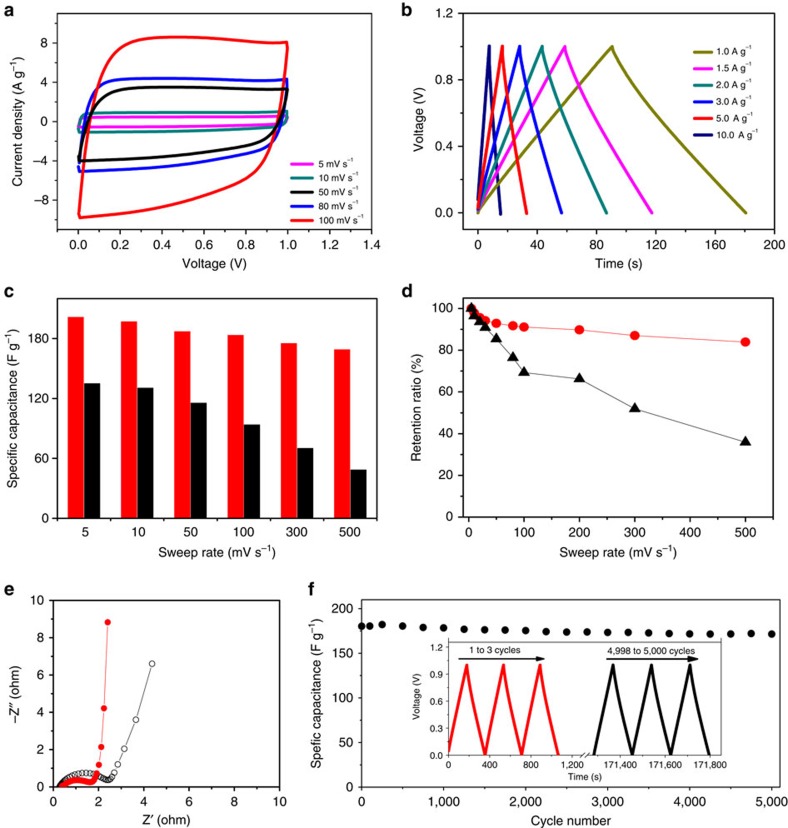
Supercapacitive performances of HCNs. (**a**) CV curves at different sweep rates and (**b**) galvanostatic charge–discharge curves under various current densities for HCN-900-10H5R. (**c**) Specific capacitances and (**d**) capacitance retention ratios of HCN-900-10H5R (red) and AC (black). (**e**) Electrochemical impedance spectra of HCN-900-10H5R (red) and AC (black). (**f**) Long-term cycle stability over 5,000 cycles for HCN-900-10H5R at current density of 1 A g^−1^; the inset shows the curves for the first and last three cycles.

**Figure 6:**
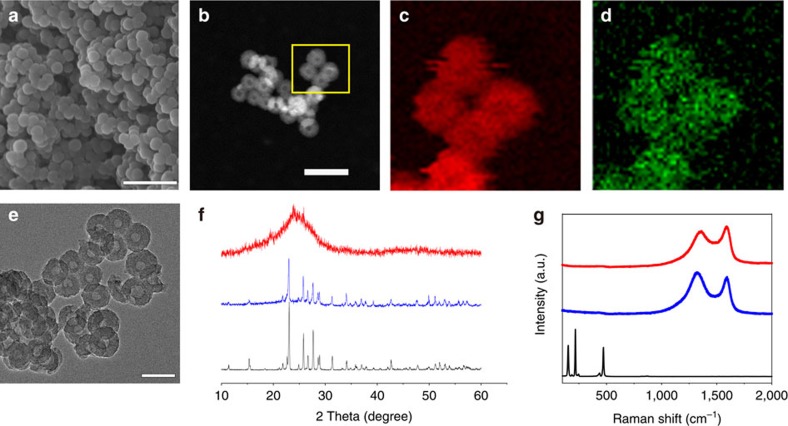
Structural characterizations of HCN/S nanocomposites. (**a**) SEM and (**e**) TEM images for HCN-900-10H5R/S; (**b**) bright-field TEM image and corresponding elemental mappings of (**c**) carbon and (**d**) sulphur for HCN-900-10H5R/S; (**f**) XRD patterns for HCN-900-10H5R/S (red), mixture of HCN-900-10H5R and S before melting infiltration (blue) and sulphur (black); (**g**) Raman spectra of HCN-900-10H5R/S (red), HCN-900-10H5R (blue) and sulphur (black). Scale bars, 500 nm (**a**), 200 nm (**b**) and 100 nm (**e**).

**Figure 7:**
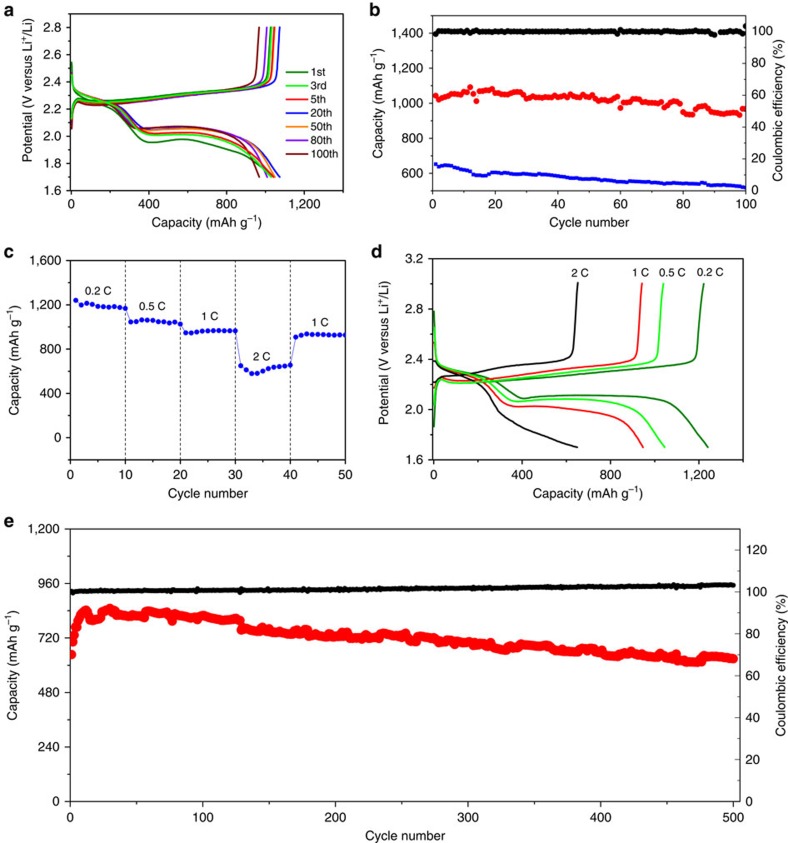
Li–S battery performances of HCN/S nanocomposites. (**a**) Discharge–charge curves recorded at different cycles for HCN-900-10H5R/S; (**b**) cycle performances at 0.5 C for HCN-900-10H5R/S (red) and mixture of HCN-900-10H5R and S before melt infiltration (blue) and coulombic efficiency (black) for HCN-900-10H5R/S; (**c**) rate performances, (**d**) discharge–charge curves at various rates and (**e**) capacity (red) and coulombic efficiency (black) over 500 cycles at 1 C after stopping the rate performance test for one month for HCN-900-10H5R/S.

It is noteworthy that the ultrahigh surface areas (for example, 3,022 m^2^ g^−1^) of our HCNs obtained here stand at the highest level among all HCNs. The S_BET_ of HCNs reported previously is generally no more than 1,800 m^2^ g^−1^ (Supplementary Table 1), and their fabrication procedures are quite complicated because they involve the formation of pre-made templates, adsorption of carbon precursors onto the surfaces of the templates, carbonization, additional activation sometimes^49^,^59^ and finally removal of templates (Fig. 1a). The ultrahigh surface area exceeds that of some commercially available activated carbons (for example, YP-50, 1,417 m^2^ g^−1^), and is slightly larger than the theoretical surface area of graphene (that is, 2,600 m^2^ g^−1^; ref. 60) and a double-sided separated graphitic sheet (2,965 m^2^ g^−1^; ref. 61). Chemical activation using KOH is a well-established method to generate highly porous carbons with SSAs up to 3,000 m^2^ g^−1^ (refs 8, 9, 23). However, the rigorous etching conditions seriously destroy the original nanostructure in most cases. For convincing comparison, KOH activation was also applied to hollow PACP carbon precursor. A comparable S_BET_ of 3,200 m^2^ g^−1^ can be achieved for the resulting activated carbon AC-PACP (Supplementary Fig. 24), with a KOH to semi-carbonized PACP ratio of 5:1. Nevertheless, the hollow nanostructure is completely destroyed owing to such extensive activation (Supplementary Fig. 25). The KOH activation treatment is really harmful to the activation equipment and environment. Another drawback of KOH activation is that a great amount of oxygen-containing groups, such as hydroxyl groups, will be introduced into activated carbon, which is detrimental to its applications where highly pure carbon is required^31^. Also, no metal species are involved in the whole preparation procedure, which is different from some nanostructured carbons from metal-containing catalytic growth. Taken together, the advantages of the current template-free and activation-free approach are self-evident, in respect of the elimination of hard templates and activating agents for simplifying synthetic protocols and, in addition, reducing environmental unfriendly waste.

Benefiting from ultrahigh SSA with the adjustable porous structure and well-defined hollow nanospherical morphology, the HCNs demonstrate unprecedented yet controlled uptake capacities towards toluene and methanol vapours at room temperature, outperforming activated carbons and many other highly porous materials. The HCNs are also superior electrode materials for supercapacitive energy storage; the well-developed small-sized nanopores in the shell enhance electric double layer for efficient charge storage, whereas the hollow nanoparticle-induced short distance significantly facilitates the ion transport capability, far exceeding the commercial activated carbons and many other porous carbons. Moreover, as cathode host materials for Li–S batteries, the HCNs demonstrate large capacity, high rate capability, high coulombic efficiency and long-term stability, because their conductive carbon shell enhances charge transfer, and their well-developed nanopores sequester the sulphur and soluble polysulphide intermediates and accommodate volume expansion of sulphur during redox cycling.

In summary, we have successfully developed a facile method without the assistance of templating and activation steps to prepare HCNs with ultrahigh surface areas and uniform nanomorphologies. This is achieved via the simple carbonization treatment of well-orchestrated PACP hollow spheres. By controlling the carbonization conditions, the nanoporous structure can be well tailored while retaining the uniform hollow nanospherical architecture. These HCNs not only demonstrate excellent adsorption properties but also perform very well as electrodes for supercapacitors and as cathode host materials for Li–S batteries. We hope that the unique combination of well-defined hollow nanospherical morphology and highly porous structures may open new opportunities to create a class of novel high-performance porous materials suitable for a variety of practical applications oriented to energy, environment, catalysis medicine and others.

## Methods

### Materials synthesis

HCNs were synthesized by direct carbonization of PACP hollow spheres without any tedious templating and activation procedures. In a typical protocol, 0.38 ml aniline and 0.29 ml pyrrole were added to 60 ml deionized water containing 0.06 g Triton X-100, forming a homogeneous solution. Then, the precooled aqueous solution of ammonium persulphate was added to the above precooled mixture for polymerization for 12 h at 0 °C. Finally, the product was washed with deionized water until the filtrate became colourless and dried under vacuum at 60 °C for 24 h. The formation of hollow structure of the PACP products could be explained briefly as follows: first, aniline and pyrrole comonomers entered Triton X-100 micelles and stayed at different locations of the micelles due to their different hydrophobic properties. Subsequently, copolymerization of aniline and pyrrole began at the micelle–water interface upon addition of oxidant, and in the meantime more hydrophobic pyrrole quickly diffused from the micellar interiors for copolymerization, thus leading to formation of hollow structure. The as-obtained PACPs were heated at designated temperatures for the desired hours with different heating rates in a furnace under a N_2_ flow to produce various targeted HCNs. The resulting HCNs were denoted as HCN-*x*-yH*z*R, where the *x* indicates the applied carbonization temperatures, *y* the carbonization times and *z* the heat rate. The applied calcination temperatures were varied from 400 to 1,000 °C with carbonization time from 3 to 20 h and heating rate of 2–10 °C min^−1^.

### Preparation of HCN/S nanocomposites

Sulphur (Aldrich, with a purity of >99.995%) and HCN were thoroughly mixed in a quartz mortar for 0.5 h to yield a black mixture. The mixture was sealed in a glass container filled with nitrogen and heated first at 115 °C with a heating rate of 1 °C min^−1^ and then to 155 °C with a heating rate of 0.5 °C min^−1^, and kept at this temperature for 10 h to ensure a complete infiltration of sulphur into HCNs, leading to formation of HCN/S nanocomposites.

### Material characterizations

The nanostructures of the samples were investigated by a Hitachi S-3400 SEM and a FEI Tecnai G2 Spirit TEM. About 100 nanospheres in a scanning electron microscope image were picked at random, and then a statistical analysis of particle size distribution was carried out. The electrical conductivity was measured by a FT-330 four-probe resistivity measuring instrument. The tapping density was measured by a FT-100DA tapping density tester. Elemental mapping images were obtained by a FEI F30 TEM. N_2_ adsorption measurement was carried out using a Micromeritics ASAP 2020 analyser at 77 K. The S_BET_ was determined by BET theory. The total pore volume (*V*_t_) was estimated from the amount adsorbed at a relative pressure *P*/*P*_0_ of ca. 0.99. The pore size distribution was analysed by original density functional theory combined with non-negative regularization and medium smoothing. The thermal stability of the samples was monitored using a thermogravimetric analysis (TGA Q50) under nitrogen flow with a heating rate of 10 °C min^−1^. XPS (ESCALab250) was used for XPS spectrum, and Raman spectrum was conducted on a Renishaw inVia Laser Micro-Raman spectrometer. A Hiden IG-3 intelligent gravimetric analyser was used to determine the adsorption capacities of the samples towards organic vapours.

### Cell fabrication and measurements for EDLC

The EDLC performances were carried out in 6 M KOH electrolyte using a symmetric sandwich-type two-electrode testing cell. The electrodes were prepared by pressing a mixture of carbon samples, polytetrafluoroethylene and commercial carbon black in the ratio of 8.5:0.5:1 onto a nickel foam current collector. The electrodes were dried at 110 °C for 8 h. A sandwich-type supercapacitor consisting of two similar sample electrodes was assembled. The electrodes and separators were soaked in the electrolyte over 8 h before each assembling. All electrochemical measurements were performed with the assembled two-electrode supercapacitors at ambient temperature. The galvanostatic charge–discharge tests were executed using an Arbin instrument. CV was carried out using an IM6ex electrochemical workstation with the sweep rates from 5 to 500 mVs^−1^. Electrochemical impedance spectroscopy (excitation signal: 5 mV and frequency range: 0.1–100,000 Hz) was carried out using an IM6ex electrochemical workstation.

### Cell fabrication and measurements for Li–S battery

Cathodes were prepared by mixing HCN/S nanocomposites as the active material, conductive carbon black (Super P Li) and polyvinylidene fluoride binder (8:1:1 by weight) in *N*-methyl-2-pyrrolidinone. The obtained slurry was spread on aluminium foil using a coater, followed by removing *N*-methyl-2-pyrrolidinone under vacuum. Cathode round plates with diameter of 12 mm were prepared. The electrolyte was composed of a 1 M bis(trifluoromethane) sulphonimide lithium salt in 1,3-dioxolane and 1,2-dimethoxyethane (1:1 by volume) with 0.5 wt% LiNO_3_ additive. CR2032 coin-type cells, which were composed of cathode, a separator of polyethylene membrane and lithium plate as anode, were assembled in an argon-filled glove box for half-cell testing. All cells were tested at room temperature. The galvanostatic charge–discharge tests were conducted on a LAND instrument (model CT2001A) with a voltage window of 1.7–2.8 V versus Li^+^/Li.

## Additional information

**How to cite this article:** Xu, F. *et al.* Facile synthesis of ultrahigh-surface-area hollow carbon nanospheres for enhanced adsorption and energy storage. *Nat. Commun.* 6:7221 doi: 10.1038/ncomms8221 (2015).

## Supplementary Material

Supplementary Information Supplementary Figures 1-25, Supplementary Tables 1-6 and Supplementary References.
