# Supplementary material for: Facile synthesis of ultrahigh-surface-area hollow carbon nanospheres for enhanced adsorption and energy storage
Source: Nat Commun. 2015 Jun 15;6:7221. doi: 10.1038/ncomms8221 (PMC4490369; doi:10.1038/ncomms8221)
Supplement: Supplementary Information — Supplementary Figures 1-25, Supplementary Tables 1-6 and Supplementary References. [file ncomms8221-s1.pdf]

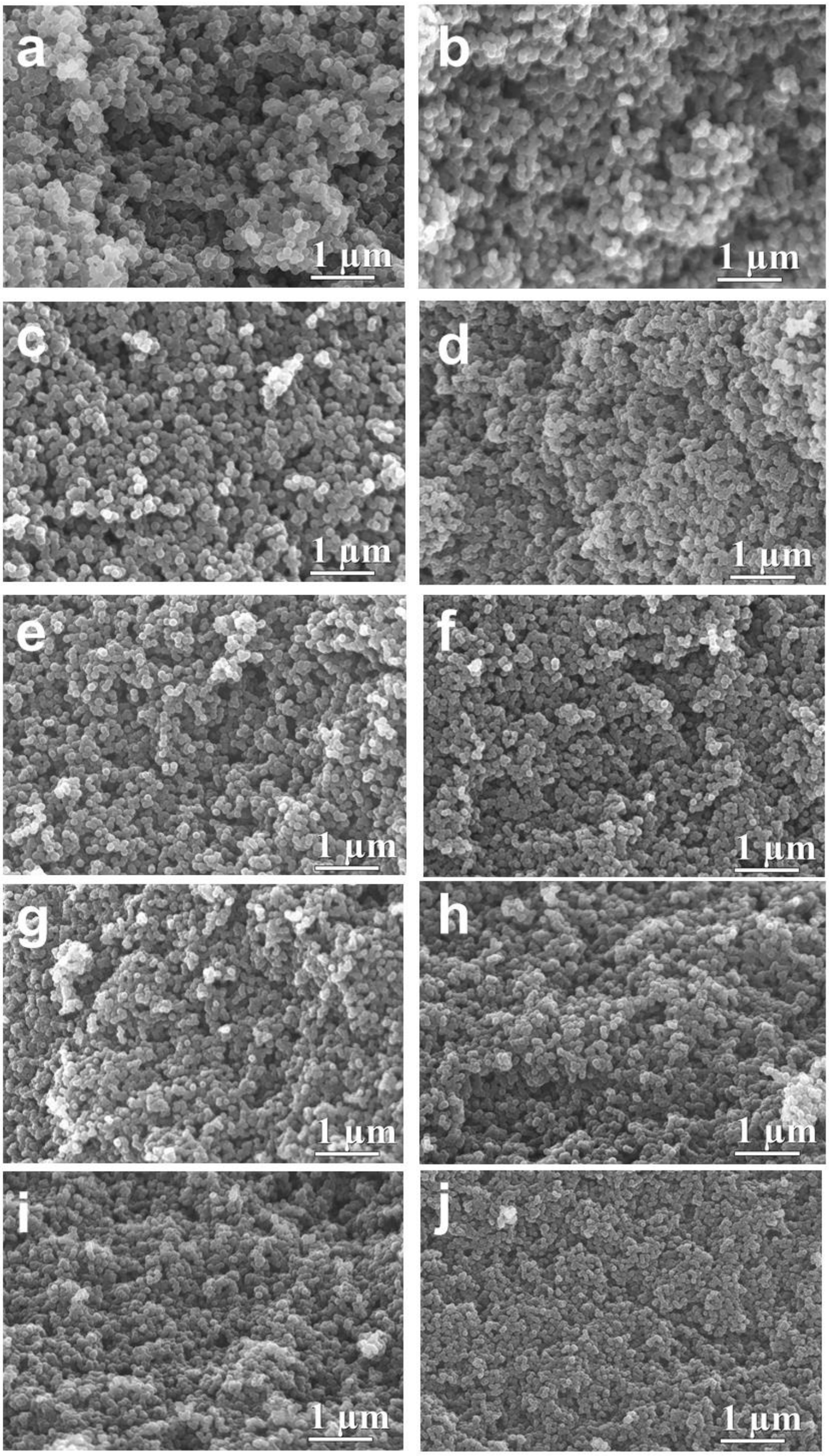

Supplementary Figure 1 | SEM images of (a) PACP, (b) HCN-400-3H2R, (c) HCN-600-3H2R, (d) HCN-800-3H2R, (e) HCN-900-3H2R, (f) HCN-900-6H2R, (g) HCN-900-10H2R, (h) HCN-900-10H5R, (i) HCN-900-10H10R, and (j) HCN-900-20H2R.

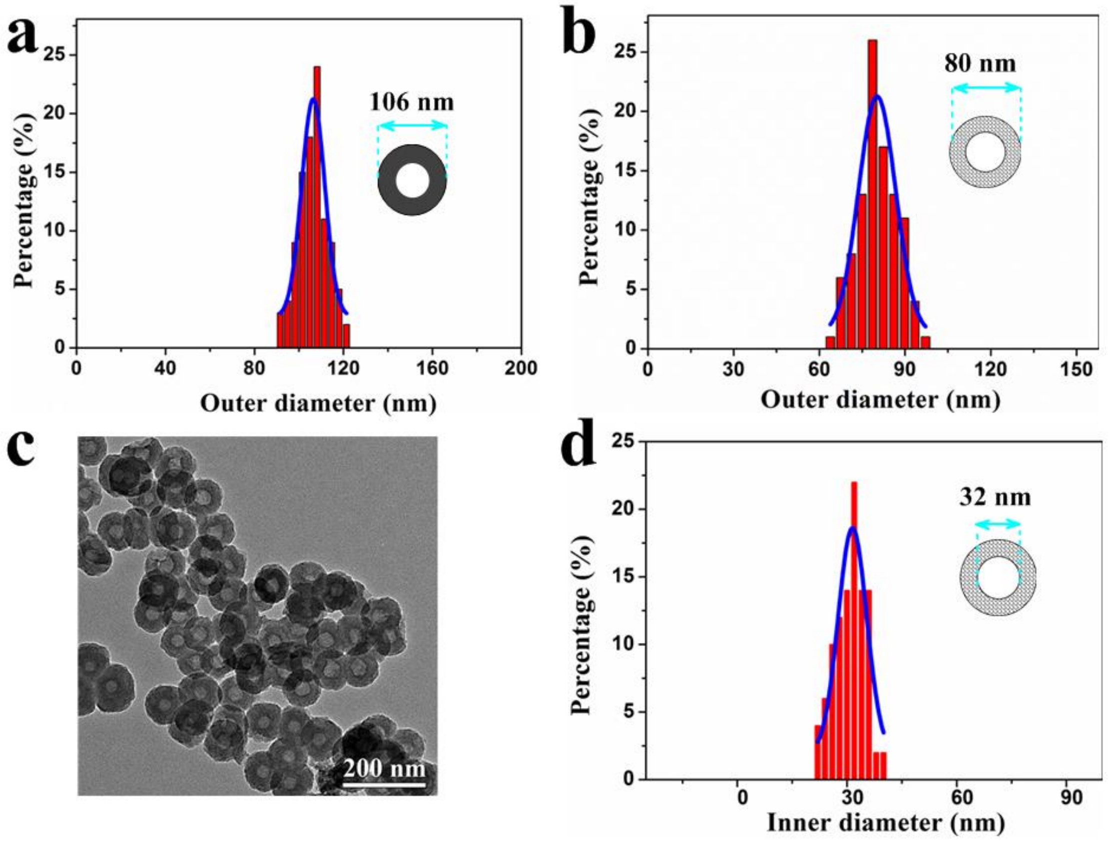

Supplementary Figure 2 | Outer diameter distribution histograms of (a) PACP and (b) HCN-900-10H5R; (c) TEM image and (d) inner diameter distribution histogram of HCN-900-10H5R.

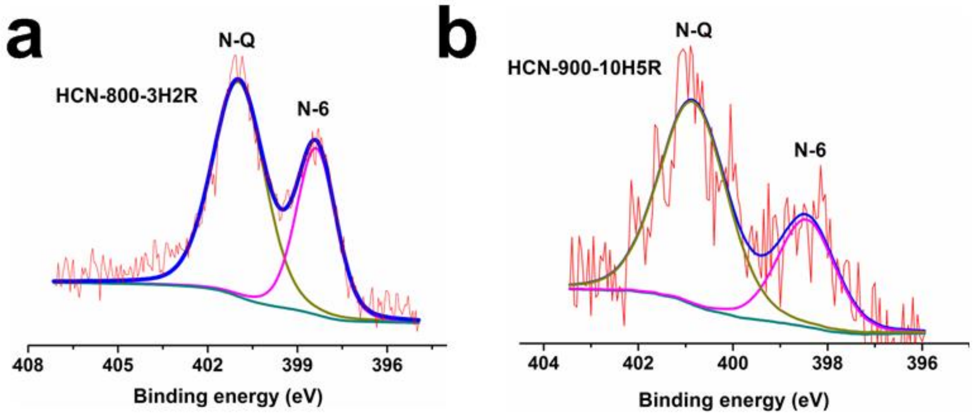

Supplementary Figure 3 | N1s spectra of HCN-800-3H2R and HCN-900-10H5R. The N1s spectra in the two HCNs can be deconvoluted into two peaks around 398.2 and 400.9 eV. The former is associated with pyridine-type N atoms (N-6) existing at the edge of the graphene sheets, and the latter is ascribed to quaternary N atoms (N-Q) incorporated in the graphene sheets.

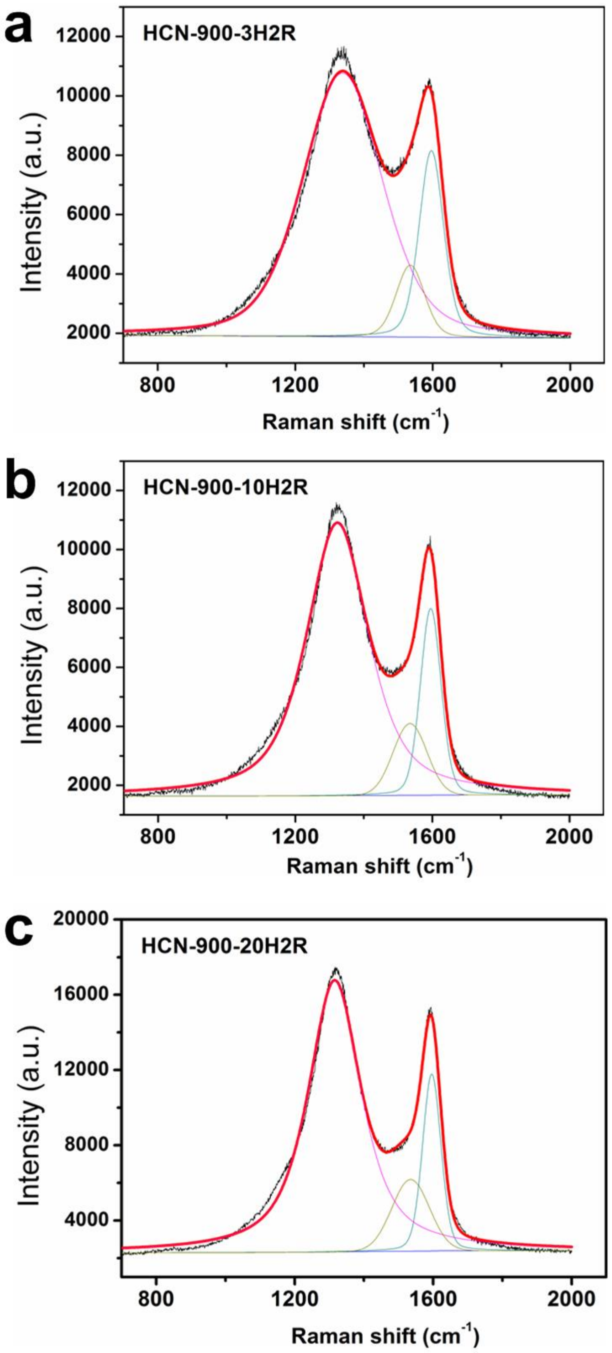

Supplementary Figure 4 | Representative Raman spectra of HCNs. In the Raman spectra, three bands around 1590, 1535, and 1330 cm^{-1}, can be deconvoluted with a Lorentzian Fit Multipeaks analysis. The band around 1590 cm^{-1} is known as the G (graphitic) mode and is attributed to "in-plane" zone-centre atomic vibrations of large graphite crystallites. The band around 1330 cm^{-1} is regarded as D (disordered) mode, owing to the phonons near the Brillouin zone boundary active in small crystallites or on the boundaries of larger crystallites. The microcrystalline planar crystal size L_a can be calculated using the empirical formula found by Tuinstra and Koenig (L_a = 4.35 I_G/I_D (nm), where I_G and I_D are integrated intensity of G peak and D peak (Supplementary Table 3), respectively). A low I_G/I_D band intensity ratio of theses HCNs indicates the generation of large amounts of defect, resulting in the presence of numerous micropores.

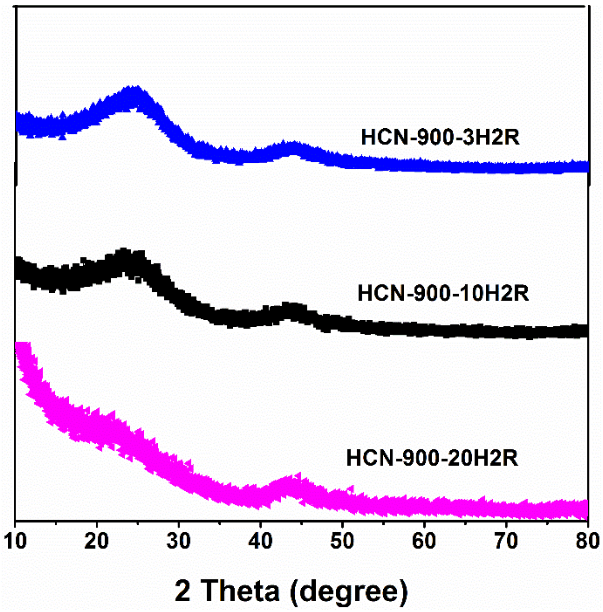

Supplementary Figure 5 | X-ray diffraction (XRD) profiles of HCNs. XRD curves display two broad peaks located at around 25 and 44 °, which are assigned to the carbon (002) and (101) diffractions, respectively. Judging from the weak and broad (002) peaks in the XRD profiles, the degree of graphitization could be low, indicating a low concentration of parallel single layers in the resulting HCN carbon frameworks, in agreement with the low microcrystalline planar crystal size obtained from Raman spectra.

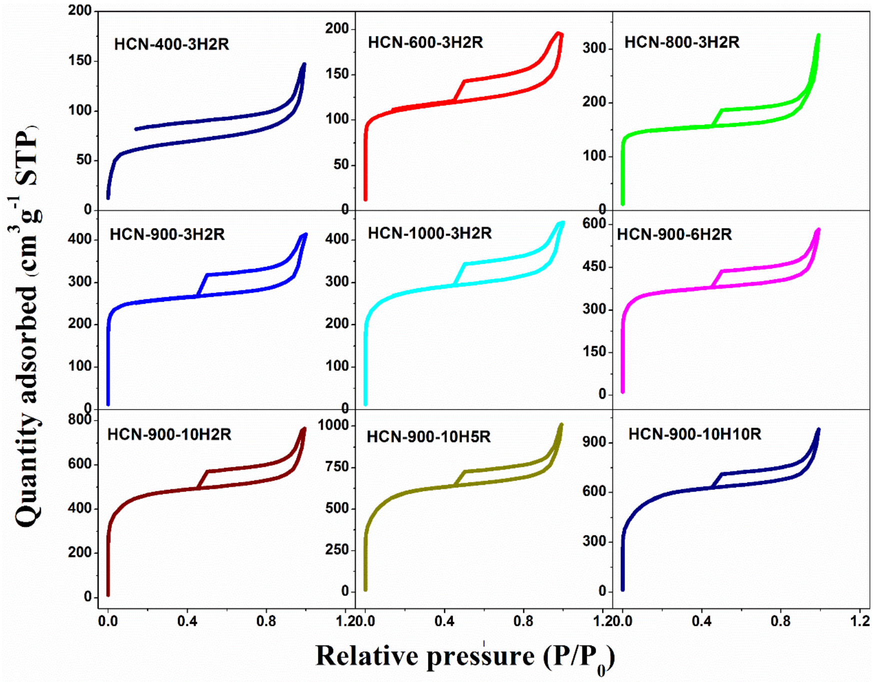

Supplementary Figure 6 | N_2 adsorption-desorption isotherms of the HCNs obtained under various carbonization conditions.

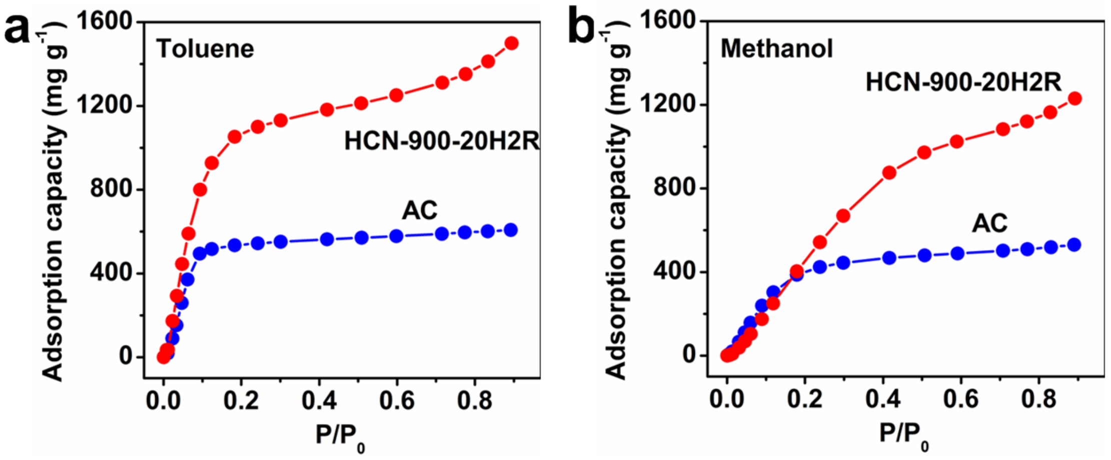

Supplementary Figure 7 | Adsorption properties toward organic vapors for the HCN-900-20H2R and commercial available activated carbon (AC).

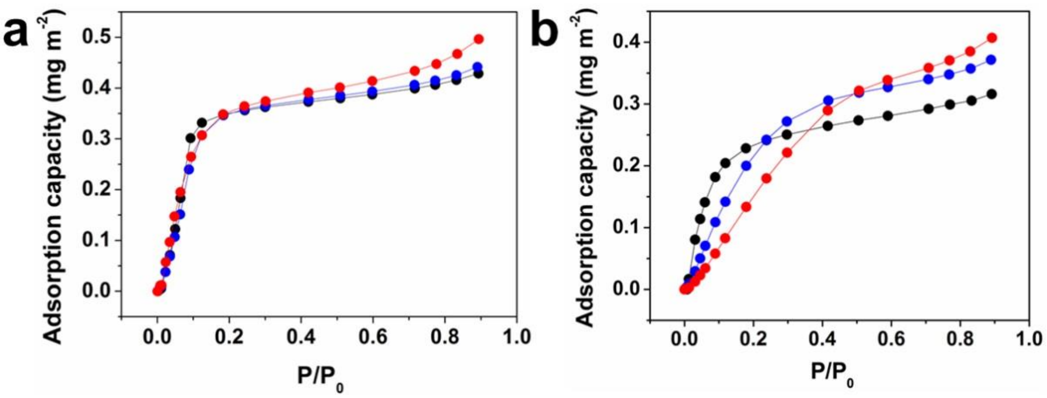

Supplementary Figure 8 | Specific adsorption capacity per unit surface area toward (a) toluene and (b) methanol vapors at various relative pressures for HCN-900-20H2R (red), HCN-900-10H5R (blue), and HCN-900-3H2R (black).

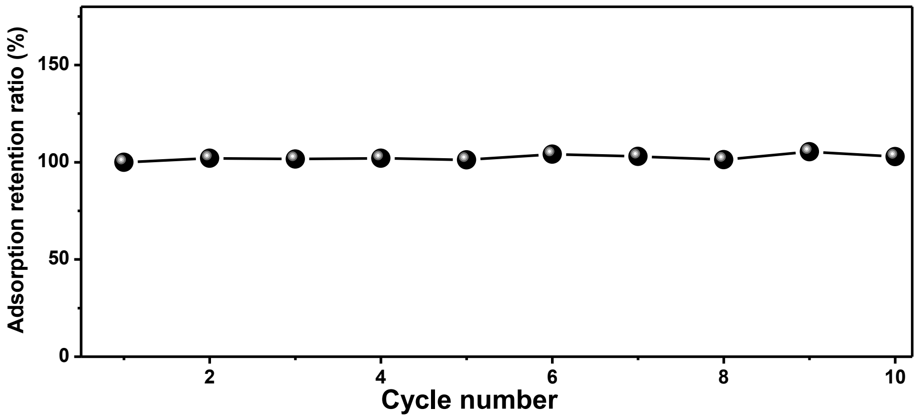

Supplementary Figure 9 | Adsorption retention ratio upon 10 cycles for HCN-900-10H5R toward toluene vapor. The adsorption retention ratio was determined by the adsorption amount divided by the adsorption amount at the first cycle.

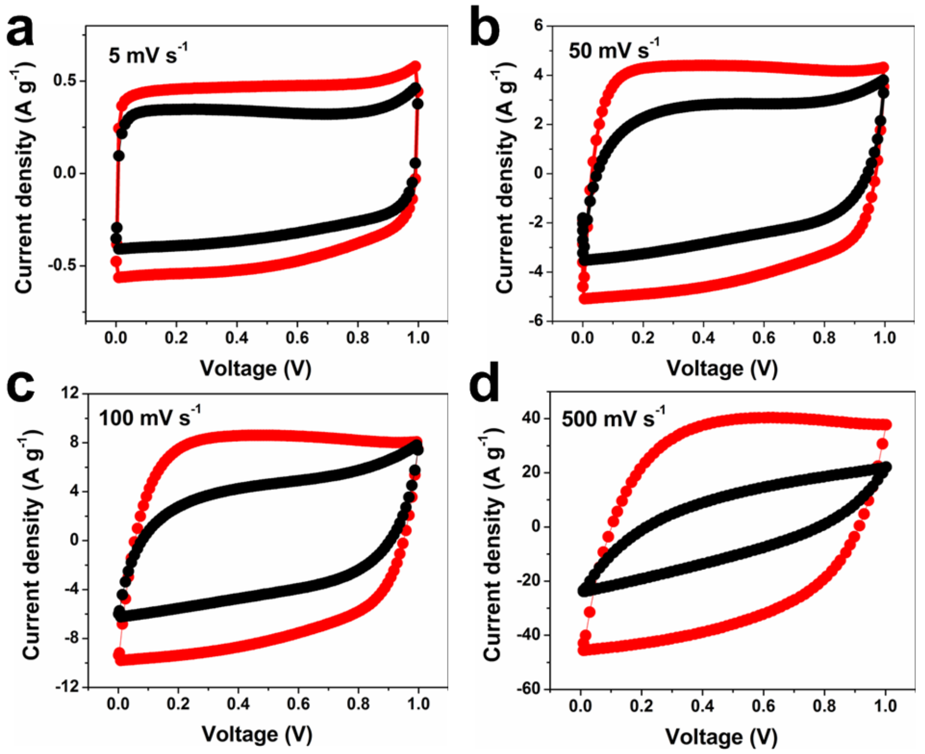

Supplementary Figure 10 | Cyclic voltammograms of HCN-900-10H5R (red) and AC (black) recorded at different sweep rates.

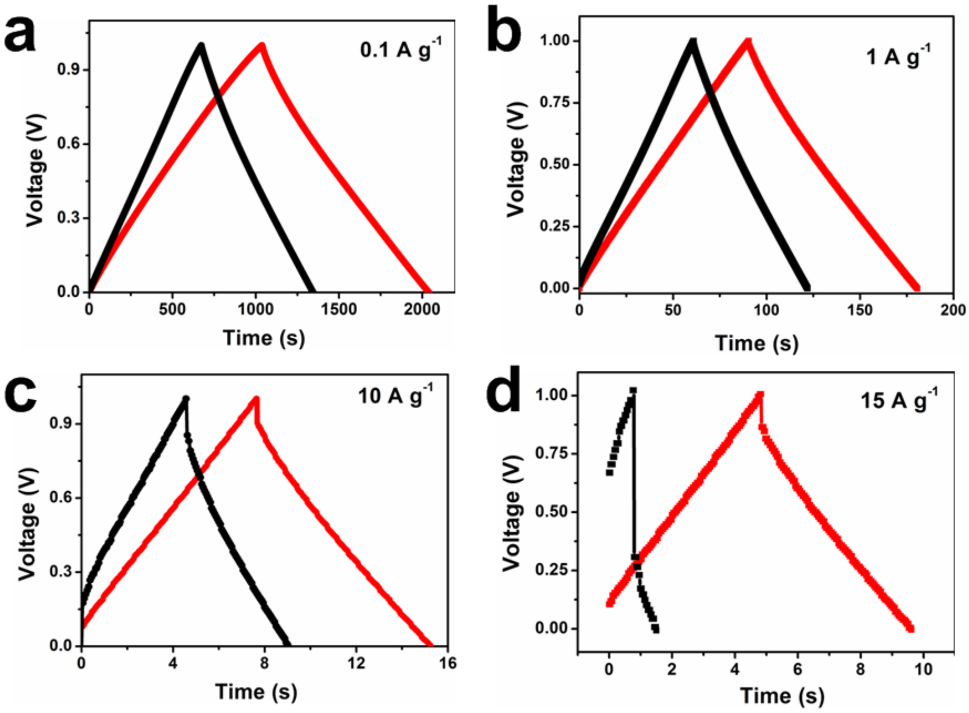

Supplementary Figure 11 | Galvanostatic charge-discharge curves of HCN-900-10H5R (red) and AC (black) recorded at different current densities.

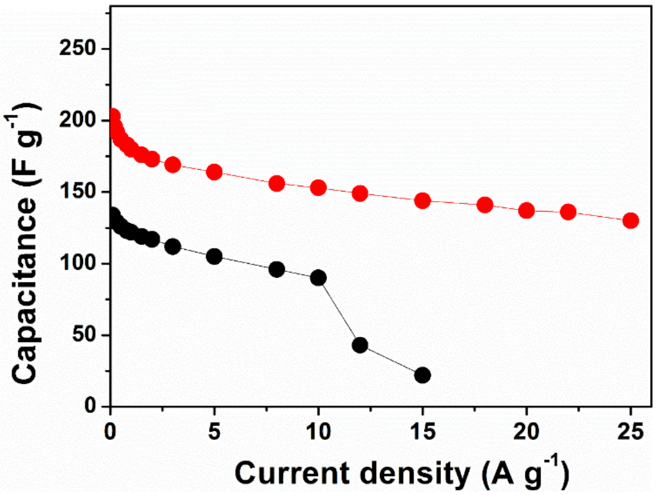

Supplementary Figure 12 | Specific capacitances of HCN-900-10H5R (red) and AC (black) under different charge-discharge current densities.

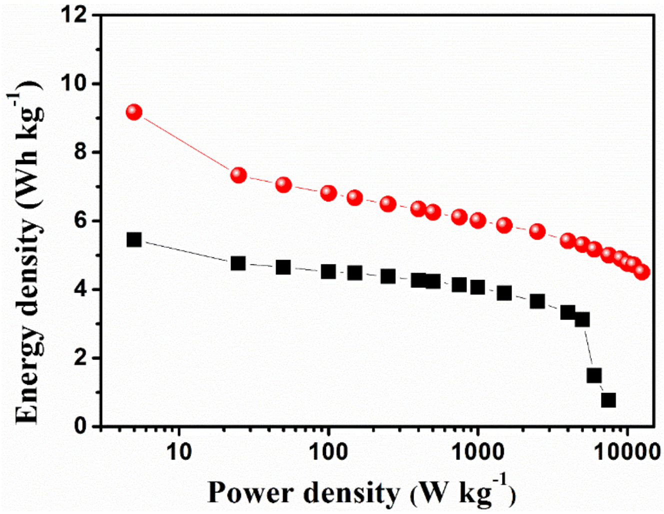

Supplementary Figure 13 | Ragone plots of HCN-900-10H5R (red) and AC (black).

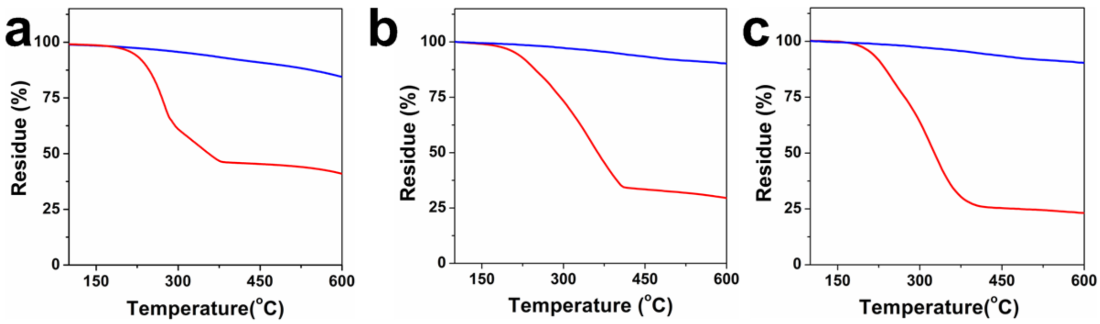

Supplementary Figure 14 | TGA curves of (a) HCN-800-3H2R (blue) and HCN-800-3H2R/S (red), (b) HCN-900-10H5R (blue) and HCN-900-10H5R/S (red), and (c) HCN-900-20H2R (blue) and HCN-900-20H2R/S (red), revealing that the sulphur content of HCN-800-3H2R/S, HCN-900-10H5R/S and HCN-900-20H2R/S is 43%, 61% and 67%, respectively.

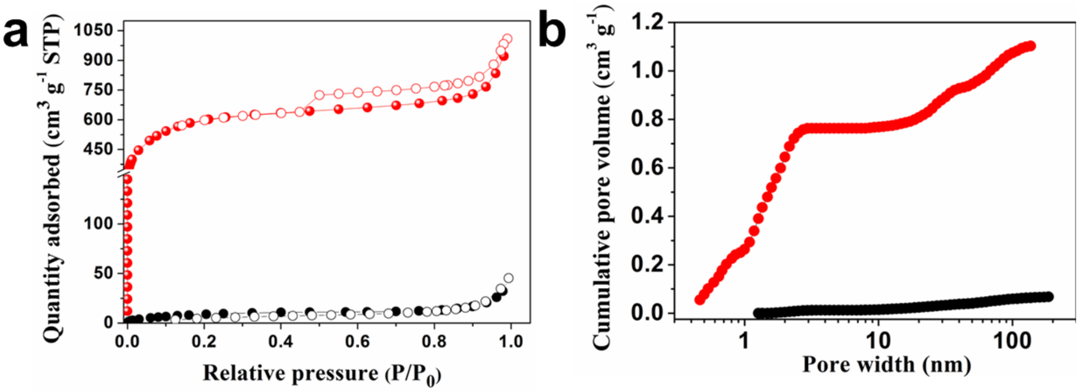

Supplementary Figure 15 | (a) Nitrogen adsorption-desorption isotherms and (b) DFT cumulative pore volume curves as a function of pore width for HCN-900-10H5R (red) and HCN-900-10H5R/S (black).

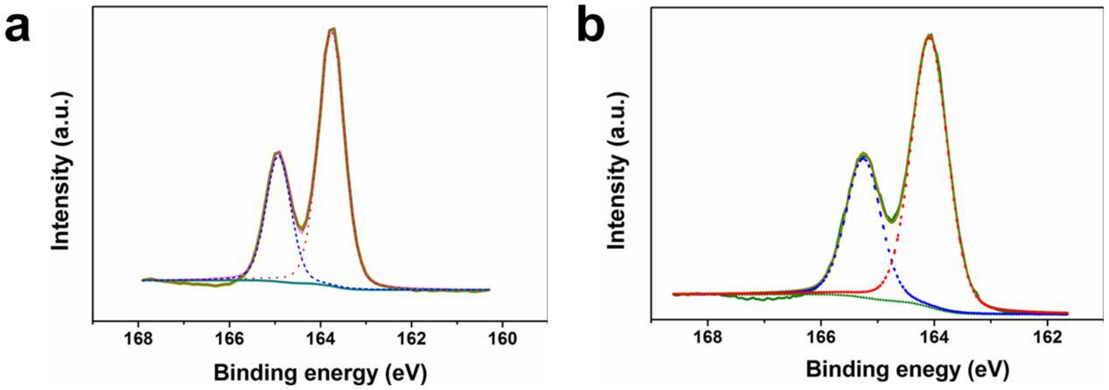

Supplementary Figure 16 | S 2p XPS spectra of (a) elemental sulphur and (b) sulphur in HCN-900-10H5R/S.

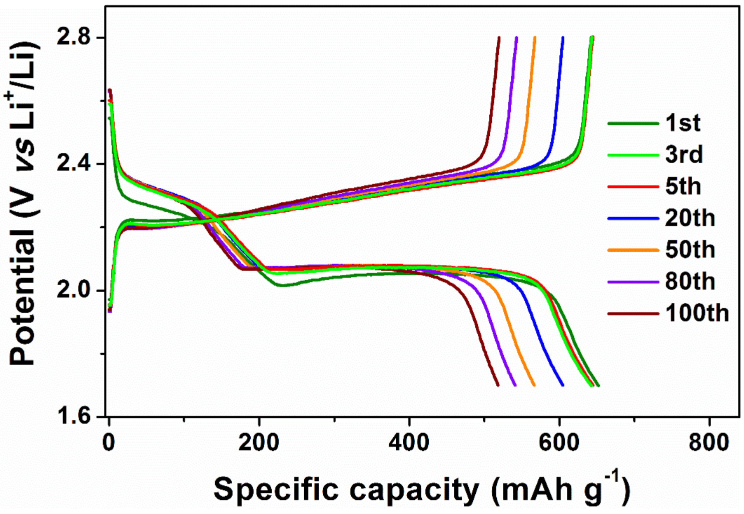

Supplementary Figure 17 | Discharge-charge curves of mixture of HCN-900-10H5R and S before melt infiltration at 0.5 C.

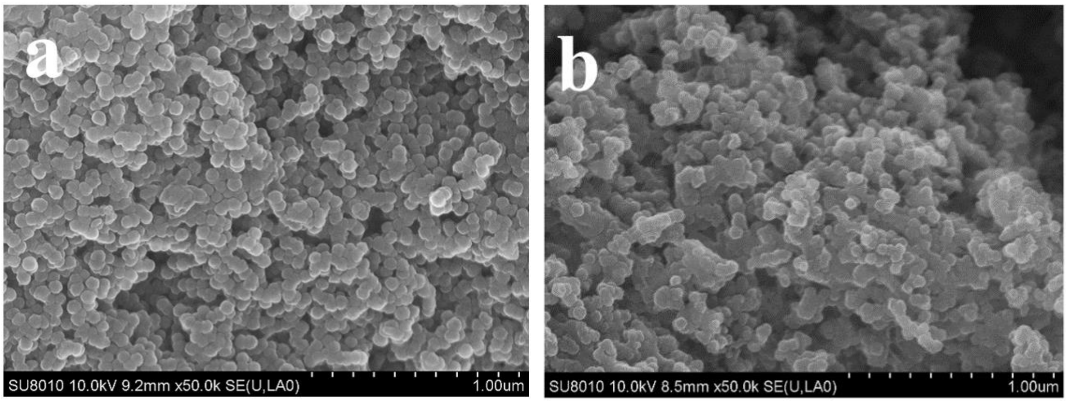

Supplementary Figure 18 | SEM images of HCN-900-10H5R/S electrode (a) before and (b) after 100 cycles.

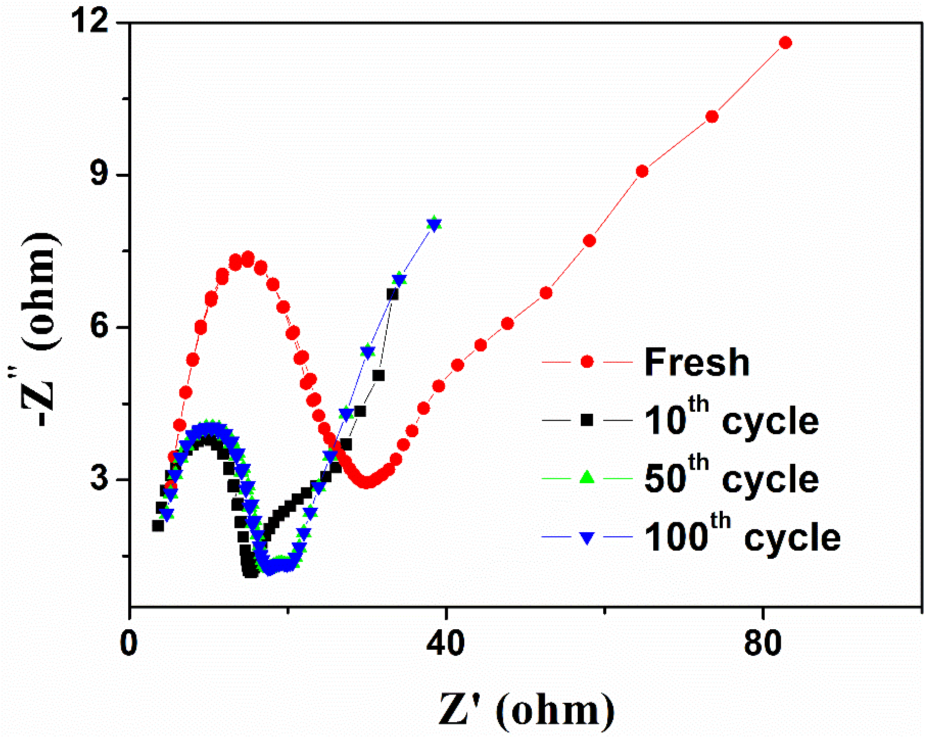

Supplementary Figure 19 | Electrochemical impedance spectra of HCN-900-10H5R/S cathode recorded at different cycles under 0.5 C rate. The impedance spectra, consisting of a depressed semicircle in the high-frequency region and an oblique line in the medium-frequency region, reflect the changes in impedance of the electrode before and after different cycles. Obviously, there are few changes in the diameter of the semicircles in the high-frequency region after 10 cycles, revealing that the electrochemical impedances do not increase dramatically. This further demonstrates the robust properties and superior conductivity of hollow carbon nanospheres for sulphur nanoconfinement. The initial large resistance can be ascribed to the electrolyte penetration process for activation.

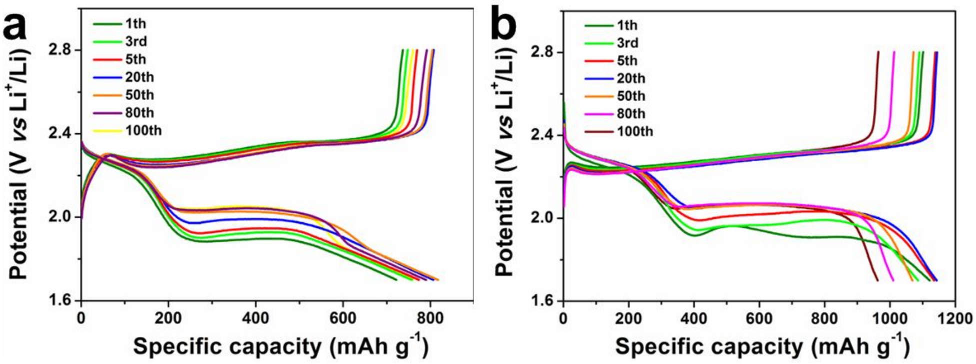

Supplementary Figure 20 | Discharge-charge curves recorded at different cycles for (a) HCN-800-3H2R/S and HCN-900-20H2R/S.

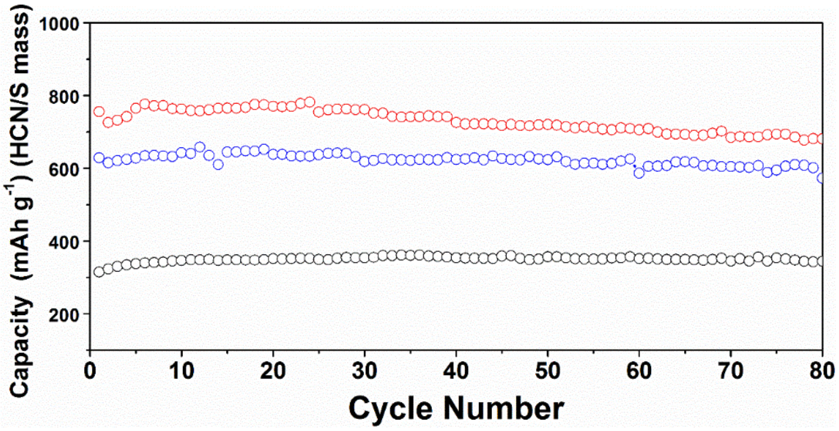

Supplementary Figure 21 | Capacities based on HCN/S composites over 80 cycles at 0.5 C for HCN-900-20H2R/S (red), HCN-900-10H5R/S (blue) and HCN-800-3H2R/S (black).

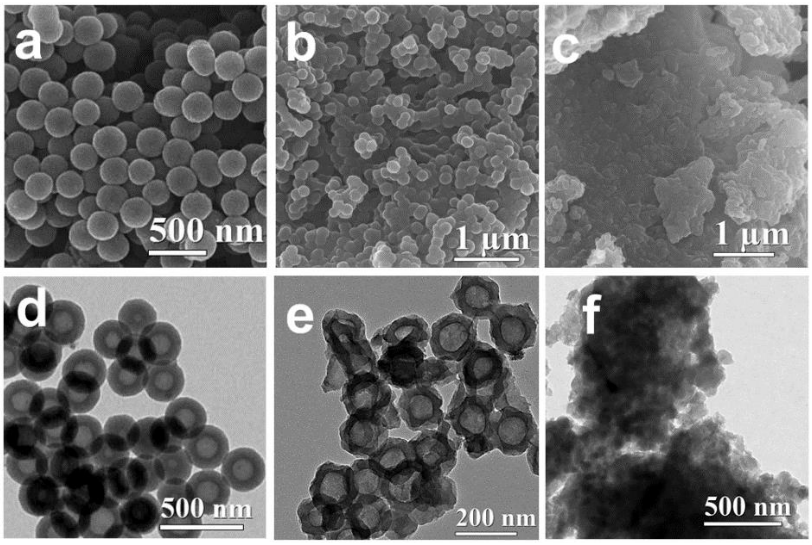

Supplementary Figure 22 | (a) SEM and (d) TEM images of crosslinked polystyrene based hollow nanosphere; (b) SEM and (e) TEM images of carbon product of crosslinked polystyrene based hollow nanosphere at 900 °C for 3 h; (c) SEM and (f) TEM images of carbon product of crosslinked polystyrene based hollow nanosphere at 900 °C for 20 h.

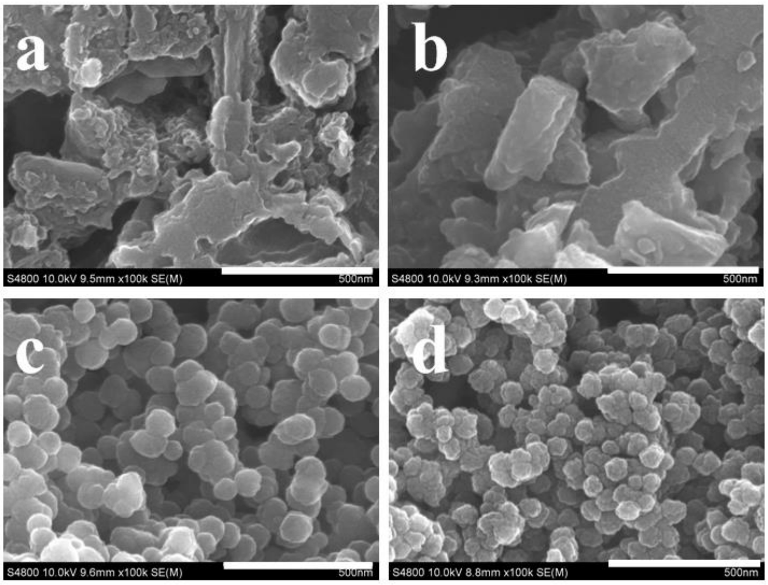

Supplementary Figure 23 | SEM images of the homo-polymeric carbon precursors by polymerization of (a) aniline and (c) pyrrole under the synthesis condition similar to the copolymerization case, and the corresponding carbon products of (b) polyaniline and (d) polypyrrole at 800 °C for 3 h. The scale bar is 500 nm.

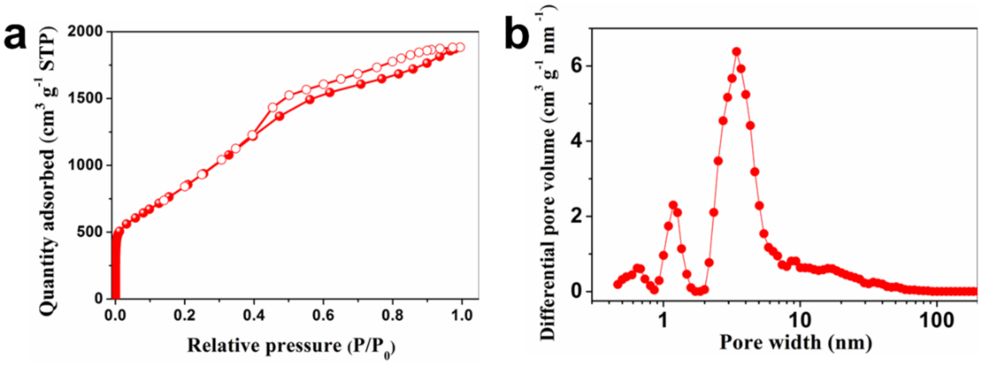

Supplementary Figure 24 | (a) N_2 adsorption-desorption isotherm and (b) DFT pore size distribution for AC-PACP.

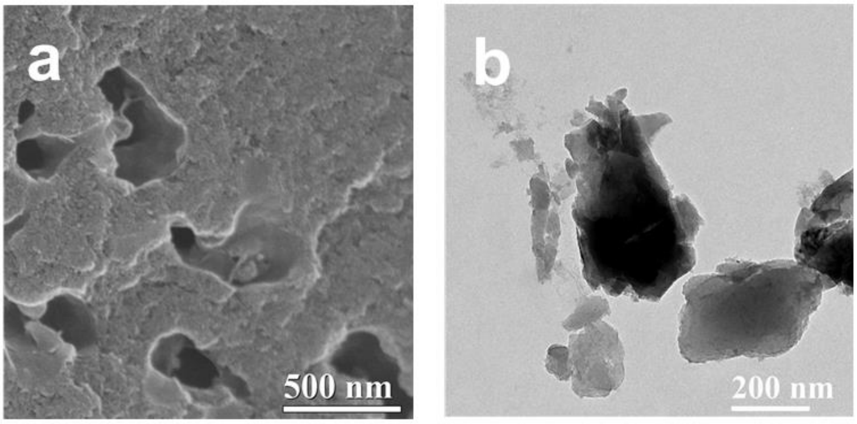

Supplementary Figure 25 | (a) SEM and (b) TEM images of AC-PACP.

Supplementary Table 1. BET surface areas, diameters, and preparation methods of the hollow carbon nanospheres summarized from literatures.

**References**	**BET surface area (m^2 g^{-1})**	**Diameter (nm)**	**Preparation methods**
1	719	760	PS templating
2	-	400	Silica templating
3	389	130	PS templating
4	658	100	Silica templating
5	970	380	Silica templating
6	604	100-440	PS templating
7	1800	200	Silica templating and KOH activation
8	1080	200	CO_2 activation
9	1520	100	Silica templating
10	648	200	Silica templating
11	996	100	Silica templating
12	748	300-500	SnO_2 hollow sphere templating
13	444	900	Lysin templating,
14	1620	440	Silica templating
15	1724	720	PS templating
16	767	220	Silica templating
17	426	330	Silica templating
18	213	550	PS templating
19	1538	400	Silica templating
20	1370	130	Silica templating
21	106	450	PMMA-EA-AA templating
22	1667	510	Silica templating

Supplementary Table 2. Parameters determined by Raman spectra of HCNs.

Sample	Peak position (cm^{-1})			Peak area			La
	D	A	G	D	A	G	
HCN-900-3H2R	1338	1535	1596	3007488	274600	638266	0.92
HCN-900-10H2R	1323	1535	1595	2754284	300197	521042	0.82
HCN-900-20H2R	1316	1535	1596	3808924	517223	686548	0.78

Supplementary Table 3. Summary of pore parameters for HCNs obtained at various carbonization conditions.

Sample	S_{BET} (m^2 g^{-1})	S_L (m^2 g^{-1})	S_{mic} (m^2 g^{-1})	V_{mic} (cm^3 g^{-1})	V_t (cm^3 g^{-1})
HCN-400-3H2R	219	296	119	0.05	0.23
HCN-600-3H2R	378	506	269	0.12	0.30
HCN-800-3H2R	504	673	411	0.19	0.50
HCN-900-3H2R	858	1144	700	0.33	0.64
HCN-1000-3H2R	936	1263	602	0.28	0.68
HCN-900-6H2R	1222	1637	927	0.43	0.90
HCN-900-10H2R	1595	2174	987	0.45	1.18
HCN-900-20H2R	3022	4225	165	0.02	2.43
HCN-900-10H5R	2095	2864	1063	0.47	1.56
HCN-900-10H10R	2050	2826	889	0.39	1.51

Note: S_{BET}, S_L, S_{mic}, V_{mic} and V_t denote BET surface area, Langmuir surface area, micropore surface area, micropore volume and total pore volume, respectively.

Supplementary Table 4. Summary of the adsorption amounts toward methanol and toluene vapors at room temperature (298 K) for various porous materials.

Sample		Adsorption amount (mg g^{-1})		References
		Methanol	Toluene	
**HCN-900-20H2R**		**1230**	**1500**	**This work**
**Activated carbon (YP-50)**		**531**	**608**	
**Porous carbons**	Activated carbon	-	640 (273K)	23
	Spherical activated carbon		460	24
	Hierarchical porous carbon microfibers	243	-	25
	Activated carbon fiber cloth	-	600 (293 K)	26
	Activated graphene aerogels	641	710	27
	Single-walled carbon nanotube (SWNT)	-	456	28
**Nanoporous polymers**	Porous polymeric nanoscale networks	741	-	29
	Ordered mesoporous polymers	439	-	29
	Microporous polymeric microsphere	574	887	30
	Porous aromatic framework (PAF-12)	289	-	31
	Porous aromatic framework (PAF-5)	934	1062	32
	Porous aromatic framework (PAF-20)	609	-	33
	Porous aromatic framework (PAF-11)	654	780	34
	Porous aromatic framework (PAF-1)	-	1357	35
	Porous conjugated polyporphyrins (CPOP-12)	766	1192	36
	Mesoporous conjugated polycarbazole (CPOP-9)	-	1355	37
**Metal-organic frameworks**	$[Zn_4O\text{-}(bdc)(bpz)_2]\cdot 4DMF\cdot 6H_2O$	100	125	38
	$[Zn_4O\text{-}(bdc)(bpz)_2]\cdot 4DMF\cdot 6H_2O$	480	510	39
	MIL-101	-	1110	40
	$PdCl_2$/MIL-101	-	1285	
	$[Cu_2(bdc)_2(DMF)]\cdot H_2O\cdot (DMF)(C_2H_5OH)_{0.5}$ (2·g)	171	-	41

Supplementary Table 5. Summary of the performances of representative porous carbon electrodes tested in aqueous electrolyte with a two-electrode symmetric cell.

**Sample**		**Current density**	**Capacitance retention ratio (%)**		
			**References**		**HCN-900-10H 5R (This work)**
**Hollow carbon spheres**	Porous nitrogen-doped hollow carbon spheres	0.5-10 A g^{-1}	55.6	18	**81.8**
	Hierarchical porous carbon hollow spheres	0.5-10 A g^{-1}	72.8	4	**81.8**
	Carbon nanocages	0.1-10 A g^{-1}	68.4	42	**75.4**
		10-100 mV s^{-1}	73.7		**93.0**
**Hierarchical porous carbons**	Hierarchical porous carbon	0.5-10 A g^{-1}	69.8	43	**81.8**
	HPC-4-800	0.025-0.2 A cm^{-2}	77.8	44	**92.9**
	3D micro-porous conducting carbon beehive	0.5-10 A g^{-1}	70.9	45	**81.8**
	HPGC	0.02-10 A g^{-1}	64.5	46	**78.0**
	Hierarchical porous carbon microfibers	2-50 mV s^{-1}	71.6	25	**88.2**
**Ordered mesoporous carbons**	Nitrogen-doped ordered nanoporous carbons	0.1-10 A g^{-1}	50	47	**75.3**
**Graphene/CNT**	3D N-doped graphene-CNT networks	0.5-5 A g^{-1}	52.8	48	**87.7**
**Others**	Nanoporous carbon	5-50 mV s^{-1}	77.9	49	**92.9**
	Nitrogen-enriched nonporous carbon	0.05-1 A g^{-1}	70.7	50	**85.3**
	PVT800	0.05-5 A g^{-1}	60	51	**77.7**
	Microporous carbon nanosphere	0.02-2 A g^{-1}	57.9	52	**82.0**
**Activated carbon**	YP-50	0.1-15 A g^{-1}	16.4	This work	**70.9**

Supplementary Table 6. Summary of cycle stability performances of representative carbon/S cathodes.

Sample		Rate	Cycle number	Retention (%)	References
**HCN-900-10H5R/S**		**0.5 C**	**100**	**93**	**This work**
		**1 C**	**500**	**97**	
**Hollow carbon nanospheres**	Double-shelled hollow carbon spheres/S	0.3 C	100	42	12
	Porous hollow carbon spheres/S	0.5 C	50	73.4	9
	Hydrothermal hollow carbon spheres/S	1 C	50	60	11
	Nitrogen-doped porous hollow carbon spheres/S	0.2 C	150	53	19
	Porous hollow carbon/S	0.5 C	100	91	10
	Activated hollow carbon nanospheres/S	0.5 C	100	89.6	7
	Hollow spherical carbon/S	0.5 C	150	60.3	21
**(Ordered) Mesoporous carbons**	CMK-3/S	0.1 C	20	80	53
	S-BMC/S	1 C	100	65.4	54
	BMC-1/S	1 C	100	55	55
	Ordered meso-microporous core-shell carbon/S	0.5 C	200	80	56
	MS/S	0.03 C	40	40	57
	Mesoporous hard carbon spherules/S	0.6 C	45	54	58
	Carbon aerogels/S	0.6 C	50	35	59
**Hierarchically porous carbons**	Hierarchically ordered porous carbon/S	0.5 C	50	89	60
		1 C	100	74	
	Hierarchically porous carbon/S	0.24 C	20	64.5	61
	Hierarchically porous carbon/S	0.5 C	100	32	62
		1 C	100	45	
	Hierarchically porous carbon/S	0.5 C	50	66	63

**Carbon nanotubes**	SDCNT-300/S	0.25 C	100	53.3	64
	N-ACNT/G@S	1 C	80	76	65
	Hollow carbon nanofiber/S	0.5 C	150	48.4	66
	Hierarchical vine-tree-like carbon nanotube/S	1 C	450	64	67
	Crab shell-template carbon/S	0.5 C	200	59	68
	MWCNT@S/NPC@PEG	0.5 C	50	73	69
**Graphenes**	Unstacked double-layer template graphene/S	1 C	200	64.7	70
	Fibrous hybrid of graphene/S	0.4 C	100	77	71
	Layered graphene-based porous carbon/S	0.5 C	100	70	72

Supplementary References

1. Fu J.W., Xu Q., Chen J.F., Chen Z.M., Huang X.B. & Tang X.Z. Controlled fabrication of uniform hollow core porous shell carbon spheres by the pyrolysis of core/shell polystyrene/cross-linked polyphosphazene composites. *Chem. Commun.* **46**, 6563-6565 (2010).
2. Liu R. *et al.* Dopamine as a carbon source: the controlled synthesis of hollow carbon spheres and yolk-structured carbon nanocomposites. *Angew. Chem. Int. Ed.* **50**, 6799-6802 (2011).
3. White R.J., Tauer K., Antonietti M. & Titirici M.M. Functional hollow carbon nanospheres by latex templating. *J. Am. Chem. Soc.* **132**, 17360-17363 (2010).
4. Han Y., Dong X.T., Zhang C. & Liu S.X. Hierarchical porous carbon hollow-spheres as a high performance electrical double-layer capacitor material. *J. Power Sources* **211**, 92-96 (2012).
5. Yang S.B., Feng X.L., Zhi L.J., Cao Q.A. & Maier J. Müllen K. Nanographene-constructed hollow carbon spheres and their favorable electroactivity with respect to lithium storage. *Adv. Mater.* **22**, 838-842 (2010).
6. Lu A.H. *et al.* Synthesis of discrete and dispersible hollow carbon nanospheres with high uniformity by using confined nanospace pyrolysis. *Angew. Chem. Int. Ed.* **50**, 11765-11768 (2011).
7. He G., Evers S., Liang X., Cuisinier M., Garsuch A. & Nazar L.F. Tailoring porosity in carbon nanospheres for lithium-sulfur battery cathodes. *ACS Nano* **7**, 10920-10930 (2013).
8. Wang G.H., Sun Q., Zhang R., Li W.C., Zhang X.Q. & Lu A.H. Weak acid-base interaction induced assembly for the synthesis of diverse hollow nanospheres. *Chem. Mater.* **23**, 4537-4542 (2011).

9. Zhang K., Zhao Q., Tao Z.L. & Chen J. Composite of sulfur impregnated in porous hollow carbon spheres as the cathode of Li-S batteries with high performance. *Nano Res.* **6**, 38-46 (2013).
10. Jayaprakash N., Shen J., Moganty S.S., Corona A. & Archer L.A. Porous hollow carbon@sulfur composites for high-power lithium-sulfur batteries. *Angew. Chem. Int. Ed.* **50**, 5904-5908 (2011).
11. Brun N., Sakaushi K., Yu L.H., Giebeler L., Eckert J. & Titirici M.M. Hydrothermal carbon-based nanostructured hollow spheres as electrode materials for high-power lithium-sulfur batteries. *Phys. Chem. Chem. Phys.* **15**, 6080-6087 (2013).
12. Zhang C.F., Wu H.B., Yuan C.Z., Guo Z.P. & Lou X.W. Confining sulfur in double-shelled hollow carbon spheres for lithium-sulfur batteries. *Angew. Chem. Int. Ed.* **51**, 9592-9595 (2012).
13. Lu A.H. *et al.* Easy Synthesis of hollow polymer, carbon, and graphitized microspheres. *Angew. Chem. Int. Ed.* **49**, 1615-1618 (2010).
14. Valle-Vigon P., Sevilla M. & Fuertes A.B. Synthesis of uniform mesoporous carbon capsules by carbonization of organosilica nanospheres. *Chem. Mater.* **22**, 2526-2533 (2010).
15. Lee H.J., Choi S. & Oh M. Well-dispersed hollow porous carbon spheres synthesized by direct pyrolysis of core-shell type metal-organic frameworks and their sorption properties. *Chem. Commun.* **50**, 4492-4495 (2014).
16. Feng S.S. *et al.* Synthesis of nitrogen-doped hollow carbon nanospheres for CO_2 capture. *Chem. Commun.* **50**, 329-331 (2014).
17. Yoon S.B., Sohn K., Kim J.Y., Shin C.H., Yu J.S. & Hyeon T. Fabrication of carbon capsules with hollow macroporous core/mesoporous shell structures. *Adv. Mater.* **14**, 19-21 (2002).
18. Han J.P., Xu G.Y., Ding B., Pan J., Dou H. & MacFarlane D.R. Porous nitrogen-doped hollow carbon spheres derived from polyaniline for high performance supercapacitors. *J. Mater. Chem. A* **2**, 5352-5357 (2014).
19. Zhou W., Xiao X., Cai M. & Yang L. Polydopamine-coated, nitrogen-doped, hollow carbon-sulfur double-layered core–shell structure for improving lithium-sulfur batteries. *Nano Lett.* **14**, 5250-5256 (2014).
20. Böttger-Hiller F. *et al.* Twin polymerization at spherical hard templates: an approach to size-adjustable carbon hollow spheres with micro- or mesoporous shells. *Angew. Chem. Int. Ed.* **52**, 6088-6091 (2013).
21. Wang Z. *et al.* Hollow spherical carbonized polypyrrole/sulfur composite cathode materials for lithium/sulfur cells with long cycle life. *J. Power Sources* **248**, 337-342 (2014).
22. Bhattacharjya D., Kim M.S., Bae T.S. & Yu J.S. High performance supercapacitor prepared from hollow mesoporous carbon capsules with hierarchical nanoarchitecture. *J. Power Sources* **244**, 799-805 (2013).
23. Lillo-Ródenas M.A., Cazorla-Amorós D., & Linares-Solano A. Behaviour of activated carbons with different pore size distributions and surface oxygen groups for benzene and toluene adsorption at low concentrations. *Carbon* **43**, 1758-1767 (2005).
24. Romero-Anaya A.J., Lillo-Ródenas M.A. & Linares-Solano A. Spherical activated carbons for low concentration toluene adsorption. *Carbon* **48**, 2625-2633 (2010).
25. Liang Y., Wu D. & Fu R. Carbon Microfibers with hierarchical porous structure from electrospun fiber-like natural biopolymer. *Sci. Rep.* **3**, 1119 (2013).

26. Luo L., Ramirez D., Rood M.J., Grevillot G., Hay K.J. & Thurston D.L. Adsorption and electrothermal desorption of organic vapors using activated carbon adsorbents with novel morphologies. *Carbon* **44**, 2715-2723 (2006).
27. Sui Z., Meng Q., Li J., Zhu J., Cui Y. & Han B. High surface area porous carbons produced by steam activation of graphene aerogels. *J. Mater. Chem. A* **2**, 9891-9898 (2014).
28. Agnihotri S., Rood M.J. & Rostam-Abadi M. Adsorption equilibrium of organic vapors on single-walled carbon nanotubes. *Carbon* **43**, 2379-2388 (2005).
29. Wu D. *et al.* Preparation of polymeric nanoscale networks from cylindrical molecular bottlebrushes. *ACS Nano* **6**, 6208-6214 (2012).
30. Feng L.J., Guo J.W. & Sun Z.Y. Spirobixanthene-based microporous polymeric microsphere for gas uptake and vapor adsorption. *Mater. Lett.* **116**, 120-122 (2014).
31. Jing X. *et al.* Targeted synthesis of micro-mesoporous hybrid material derived from octaphenylsilsesquioxane building units. *Micropor. Mesopor. Mat.* **165**, 92-98 (2013).
32. Ren H. *et al.* Synthesis of a porous aromatic framework for adsorbing organic pollutants application. *J. Mater. Chem.* **21**, 10348-10353 (2011).
33. Yan Z.J., *et al.* Construction and sorption properties of pyrene-based porous aromatic frameworks. *Micropor. Mesopor. Mat.* **173**, 92-98 (2013).
34. Yuan Y. *et al.* Targeted synthesis of a porous aromatic framework with a high adsorption capacity for organic molecules. *J. Mater. Chem.* **21**, 13498-13502 (2011).
35. Ben T. *et al.* Targeted synthesis of a porous aromatic framework with high stability and exceptionally high surface area. *Angew. Chem. Int. Ed.* **48**, 9457-9460 (2009).
36. Feng L.J., Chen Q., Zhu J.H., Liu D.P., Zhao Y.C. & Han B.H. Adsorption performance and catalytic activity of porous conjugated polyporphyrins via carbazole-based oxidative coupling polymerization. *Polym. Chem.* **5**, 3081-3088 (2014).
37. Chen Q., Liu D.P., Zhu J.H. & Han B.H. Mesoporous conjugated polycarbazole with high porosity via structure tuning. *Macromolecules.* **47**, 5926-5931 (2014).
38. Lin X. *et al.* A porous framework polymer based on a Zinc(II) 4,4‘-bipyridine-2,6,2‘,6‘-tetracarboxylate: synthesis, structure, and “Zeolite-Like” behaviors. *J. Am. Chem. Soc.* **128**, 10745-10753 (2006).
39. Hou L., Lin Y.Y. & Chen X.M. Porous metal-organic framework based on μ4-oxo tetrazinc clusters: sorption and guest-dependent luminescent properties. *Inorg. Chem.* **47**, 1346-1351 (2008).
40. Qin W.P., Cao W.X., Liu H.L., Li Z. & Li Y.W. Metal-organic framework MIL-101 doped with palladium for toluene adsorption and hydrogen storage. *RSC Adv.* **4**, 2414-2420 (2014).
41. Xue D., Lin Y., Cheng X. & Chen X. A tetracarboxylate−bridged dicopper(II) paddle-wheel-based 2-D porous coordination polymer with gas sorption properties. *Cryst. Growth Des.* **7**, 1332-1336 (2007).
42. Xie K. *et al.* Carbon nanocages as supercapacitor electrode materials. *Adv. Mater.* **24**, 347-352 (2012).
43. Xu F. *et al.* Fast ion transport and high capacitance of polystyrene-based hierarchical porous carbon electrode material for supercapacitors. *J. Mater. Chem.* **21**, 1970-1976 (2011).
44. Xing W. *et al.* Hierarchical porous carbons with high performance for supercapacitor electrodes. *Carbon* **47**, 1715-1722 (2009).
45. Puthusseri D., Aravindan V., Madhavi S. & Ogale S. 3D micro-porous conducting carbon

beehive by single step polymer carbonization for high performance supercapacitors: the magic of in situ porogen formation. *Energy Environ. Sci.* **7**, 728-735 (2014).

46. Wang D.W., Li F., Liu M., Lu G.Q. & Cheng H.M. 3D aperiodic hierarchical porous graphitic carbon material for high-rate electrochemical capacitive energy storage. *Angew. Chem. Int. Ed.* **47**, 373-376 (2008)
47. Liang Y., Liu H., Li Z., Fu R. & Wu D. In situ polydopamine coating-directed synthesis of nitrogen-doped ordered nanoporous carbons with superior performance in supercapacitors. *J. Mater. Chem. A* **1**, 15207-15211 (2013).
48. You B., Wang L.L., Yao L. & Yang J. Three dimensional N-doped graphene-CNT networks for supercapacitor. *Chem. Commun.* **49**, 5016-5018 (2013).
49. Liu B., Shioyama H., Akita T. & Xu Q. Metal-organic framework as a template for porous carbon synthesis. *J. Am. Chem. Soc.* **130**, 5390-5391 (2008).
50. Hulicova-Jurcakova D., Kodama M., Shiraishi S., Hatori H., Zhu Z.H. & Lu G.Q. Nitrogen-enriched nonporous carbon electrodes with extraordinary supercapacitance. *Adv. Funct. Mater.* **19**, 1800-1809 (2009).
51. Xu B., Wu F., Chen S., Zhou Z., Cao G. &Yang Y. High-capacitance carbon electrode prepared by PVDC carbonization for aqueous EDLCs. *Electrochim. Acta* **54**, 2185-2189 (2009).
52. Li Z., Wu D., Liang Y., Fu R. & Matyjaszewski K. Synthesis of well-defined microporous carbons by molecular-scale templating with polyhedral oligomeric silsesquioxane moieties. *J. Am. Chem. Soc.* **136**, 4805-4808 (2014).
53. Ji X.L., Lee K.T. & Nazar L.F. A highly ordered nanostructured carbon-sulphur cathode for lithium-sulphur batteries. *Nat. Mater.* **8**, 500-506 (2009).
54. Schuster J. *et al.* Spherical ordered mesoporous carbon nanoparticles with high porosity for lithium-sulfur batteries. *Angew. Chem. Int. Ed.* **51**, 3591-3595 (2012).
55. He G., Ji X. & Nazar L. High "C" rate Li-S cathodes: sulfur imbibed bimodal porous carbons. *Energy Environ. Sci.* **4**, 2878-2883 (2011).
56. Li Z. *et al.* A highly ordered meso@microporous carbon-supported sulfur@smaller sulfur core-shell structured cathode for Li-S batteries. *ACS Nano* **8**, 9295-9303 (2014).
57. Wang J. *et al.* Sulfur-mesoporous carbon composites in conjunction with a novel ionic liquid electrolyte for lithium rechargeable batteries. *Carbon* **46**, 229-235 (2008).
58. Kim J., Lee D.J., Jung H.G., Sun Y.K., Hassoun J. & Scrosati B. An advanced lithium-sulfur battery. *Adv. Funct. Mater.* **23**, 1076-1080 (2013).
59. Zhang Z. *et al.* 3D interconnected porous carbon aerogels as sulfur immobilizers for sulfur impregnation for lithium-sulfur batteries with high rate capability and cycling stability. *Adv. Funct. Mater.* **24**, 2500-2509 (2014).
60. Ding B., Yuan C.Z., Shen L.F., Xu G.Y., Nie P. & Zhang X.G. Encapsulating sulfur into hierarchically ordered porous carbon as a high-performance cathode for lithium-sulfur batteries. *Chem-Eur. J.* **19**, 1013-1019 (2013).
61. Xia W., Qiu B., Xia D.G., Zou R.Q. & Facile preparation of hierarchically porous carbons from metal-organic gels and their application in energy storage. *Sci. Rep.* **3**, 1935 (2013).
62. Xu G.Y., Ding B., Nie P., Shen L.F., Dou H. & Zhang X.G. Hierarchically porous carbon encapsulating sulfur as a superior cathode material for high performance lithium-sulfur batteries. *ACS Appl. Mater. Inter.* **6**, 194-199 (2014).

63. Xu G.Y., Ding B., Shen L.F., Nie P., Han J.P. & Zhang X.G. Sulfur embedded in metal organic framework-derived hierarchically porous carbon nanoplates for high performance lithium-sulfur battery. *J. Mater. Chem. A* **1**, 4490-4496 (2013).
64. Guo J.C., Xu Y.H. &Wang C.S. Sulfur-impregnated disordered carbon nanotubes cathode for lithium-sulfur batteries. *Nano Lett.* **11**, 4288-4294 (2011).
65. Tang C. *et al.* Nitrogen-doped aligned carbon nanotube/graphene sandwiches: facile catalytic growth on bifunctional natural catalysts and their applications as scaffolds for high-rate lithium-sulfur batteries. *Adv. Mater.* **26**, 6100-6105 (2014).
66. Zheng G.Y., Yang Y., Cha J.J., Hong S.S. & Cui Y. Hollow carbon nanofiber-encapsulated sulfur cathodes for high specific capacity rechargeable lithium batteries. *Nano Lett.* **11**, 4462-4467 (2011).
67. Zhao M.Q. *et al.* Hierarchical vine-tree-like carbon nanotube architectures: in-situ CVD self-assembly and their use as robust scaffolds for lithium-sulfur batteries. *Adv. Mater.*, **26**, 7051-7058 (2014).
68. Yao H.B. *et al.* Crab shells as sustainable templates from nature for nanostructured battery electrodes. *Nano Lett.* **13**, 3385-3390 (2013).
69. Li Z. *et al.* A dual coaxial nanocable sulfur composite for high-rate lithium-sulfur batteries. *Nanoscale* **6**, 1653-1660 (2014).
70. Zhao M.Q. *et al.* Unstacked double-layer templated graphene for high-rate lithium-sulphur batteries. *Nat. Commun.* **5**, 3410 (2014).
71. Zhou G.M. *et al.* Fibrous hybrid of graphene and sulfur nanocrystals for high-performance lithium-sulfur batteries. *ACS Nano* **7**, 5367-5375 (2013).
72. Yang X., Zhang L., Zhang F., Huang Y. & Chen Y. Sulfur-infiltrated graphene-based layered porous carbon cathodes for high-performance lithium-sulfur batteries. *ACS Nano* **8**, 5208-5215 (2014).
